# On-Resin Assembly of Macrocyclic Inhibitors of *Cryptococcus neoformans* May1: A Pathway to Potent Antifungal Agents

**DOI:** 10.1021/acs.jmedchem.5c00396

**Published:** 2025-04-22

**Authors:** Robin Kryštůfek, Václav Verner, Pavel Šácha, Martin Hadzima, Filip Trajhan, Jana Starková, Eva Tloušt’ová, Alexandra Dvořáková, Adam Pecina, Jiří Brynda, Karel Chalupský, Miroslav Hájek, Michael J. Boucher, Pavel Majer, Jan Řezáč, Hiten D. Madhani, Charles S. Craik, Jan Konvalinka

**Affiliations:** † Institute of Organic Chemistry and Biochemistry of the Czech Academy of Sciences, Flemingovo n. 2, Prague 6 16610, Czech Republic; ‡ Department of Physical and Macromolecular Chemistry, Faculty of Science, Charles University, Hlavova 8, Prague 2 12843, Czech Republic; § Department of Biochemistry, Faculty of Science, Charles University, Hlavova 8, Prague 2 12843, Czech Republic; ∥ Department of Organic Chemistry, Faculty of Science, Charles University, Hlavova 8, Prague 2 12843, Czech Republic; ⊥ Institute of Molecular Genetics of the Czech Academy of Sciences, Vídeňská 1083, Prague 4 14220, Czech Republic; # Department of Biochemistry & Biophysics, University of California San Francisco, UCSF Genentech Hall, 600 16th St Rm N374, San Francisco, California 94158, United States; ¶ Department of Pharmaceutical Chemistry, University of California San Francisco, UCSF Genentech Hall, 600 16th St Rm S512, San Francisco, California 94158, United States

## Abstract

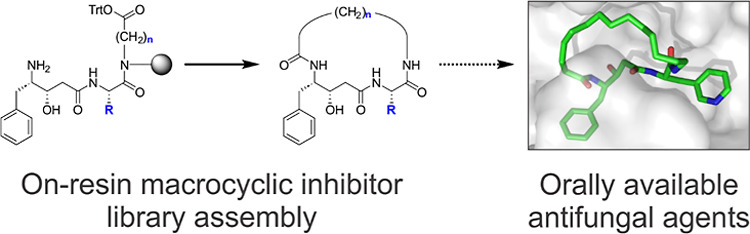

Macrocyclic inhibitors have emerged as a privileged scaffold in medicinal chemistry, offering enhanced selectivity, stability, and pharmacokinetic profiles compared to their linear counterparts. Here, we describe a novel, on-resin macrocyclization strategy for the synthesis of potent inhibitors targeting the secreted protease Major Aspartyl Peptidase 1 in *Cryptococcus neoformans*, a pathogen responsible for life-threatening fungal infections. By employing diverse aliphatic linkers and statine-based transition-state mimics, we constructed a focused library of 624 macrocyclic compounds. Screening identified several subnanomolar inhibitors with desirable pharmacokinetic and antifungal properties. Lead compound **25** exhibited a *K*_i_ of 180 pM, significant selectivity against host proteases, and potent antifungal activity in culture. The streamlined synthetic approach not only yielded drug-like macrocycles with potential in antifungal therapy but also provided insights into structure–activity relationships that can inform broader applications of macrocyclization in drug discovery.

## Introduction

Macrocyclic compounds have garnered significant attention as privileged motifs in drug discovery due to their unique ability to combine structural rigidity with functional diversity.^1−3^ Unlike linear molecules, macrocycles often display enhanced target specificity, making them attractive candidates for addressing challenging drug targets. Advances in macrocycle screening technologies, including DNA-encoded libraries,^4^ display techniques,^5,6^ and bicyclic peptide platforms^7^ have significantly expanded the accessible chemical space for discovering high-affinity binders. These methodologies facilitate rapid identification of macrocyclic inhibitors with diverse structural features and bioactive properties. Macrocycles frequently occupy beyond-Rule-of-5 (bRo5) chemical space, bridging the gap between small molecules and biologics while retaining favorable pharmacokinetics and cell permeability.^8^ Moreover, their inherent preorganization reduces entropic penalties upon binding,^9,10^ while their ability to occupy extensive surface areas enhances binding affinity and selectivity.^2,11^

Despite their advantages, macrocycle synthesis remains challenging due to inefficient cyclization protocols and limited scaffold diversity. Traditional solution-phase methods, such as high-dilution conditions and chemoenzymatic approaches, are impractical for high-throughput synthesis.^11−13^ Moreover, the inclusion of synthetic handles required for cyclization on resin can introduce undesirable physicochemical properties or additional metabolic modification sites, limiting the utility of the resulting compounds, particularly when transitioning to in vivo models.^2,14^ To address these issues, innovative solid-phase approaches enabling efficient, handle-free cyclization with broad chemical diversity are essential.

In this study, we applied a novel on-resin macrocyclization approach to generate inhibitors targeting major aspartyl peptidase 1 (May1), a secreted protease required for low pH survival and virulence of *Cryptococcus neoformans*.^15^ This opportunistic fungal pathogen is responsible for severe infections particularly in immunocompromised individuals, with an estimated annual global death toll of 118,000.^16^

May1 belongs to the peptidase A1 family and exhibits high structural homology with other secreted fungal proteases, such as secreted aspartyl proteinases (SAPs) from *Candida albicans*.^17^ Its role in *C. neoformans* virulence has been demonstrated in a mouse infection model, where may1Δ deletion strains exhibited significantly attenuated pathogenicity, leading to a more than 2-fold increase in survival time.^15^ Motivated by the therapeutic relevance of May1 as an antifungal target, we previously conducted structure-guided combinatorial screening to probe its binding preferences using a diverse set of linear scaffolds, which led to the discovery of a potent peptide inhibitor with a *K*_i_ of 12 nM.^17^ However, the development inhibitors with both increased potency and drug-like properties, such as metabolic stability and favorable pharmacokinetics, has remained elusive.

Our synthetic strategy focused on addressing the dual challenges of macrocycle assembly and inhibitor optimization. By employing aliphatic amino acids and phenylstatine-based transition-state mimics, we constructed a focused combinatorial library of 624 macrocyclic inhibitors, enabling comprehensive SAR analysis. Herein, we detail the design and synthesis of this library and demonstrate the potential of macrocyclization as a tool for enhancing the drug-like properties of inhibitors. These findings not only contribute to the development of May1 inhibitors but also underscore the broader applicability of on-resin macrocyclization in drug discovery.

## Results

### On-Resin Macrocyclic Inhibitor Library Assembly

**Scheme 1:**
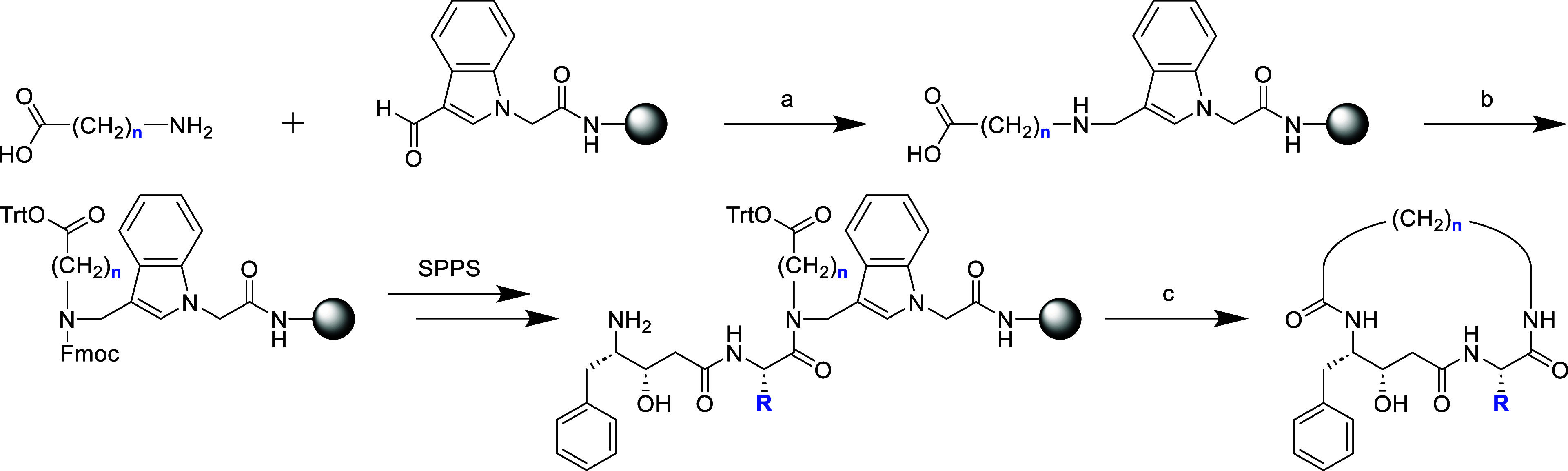
On-Resin Assembly of Macrocyclic Inhibitors of May1 Individual steps are separated by washing before the resin is transferred to the next condition. (a) 1. 3 Å sieves, AcOH/MeOH, 37 °C, 18 h; 2. NaBH_3_CN, AcOH/MeOH, RT, 18 h. (b) 1. Fmoc-Succinimide, DIEA, DMF, RT, 2 h; 2. TrtCl, DIEA, DCM, RT, 18 h. (c) 1. AcOH/MeOH/TIS 80:15:5, RT, 1 h; 2. HCTU, DIEA, DMF, RT, 24 h; 3. TFA/water 95:5, RT, 2 h.

Assembly of macrocyclic peptide inhibitors poses several synthetic challenges, particularly in achieving efficient cyclization and maintaining the drug-like properties of the resulting compounds. To address these, we developed an innovative on-resin macrocyclization strategy tailored for parallel synthesis (Scheme 1). This method circumvents limitations of solution-phase methods, such as the need for high dilution conditions or slow reagent addition to minimize intermolecular reactions,^11^ which are impractical for high-throughput synthesis.

Our approach utilizes indolyl resin as a solid-phase scaffold for macrocyclization. Initial loading of aliphatic amino acids (C4–C16, or *n* = 3–15 in Scheme 1) onto the resin required optimization due to their poor solubility in conventional reductive amination solvents such as dichloromethane and tetrahydrofuran. Glacial acetic acid proved to be an effective solvent, facilitating Schiff base formation in combination with molecular sieves as a dehydration agent (Figure S1a), which increased efficiency 3-fold compared to the previously reported dehydration agent trimethyl orthoformate (Figure S1b).^18^

To ensure orthogonality and compatibility with subsequent synthetic steps, we protected the carboxyl groups with trityl (Trt) groups. Although Trt groups are not fully orthogonal compared to other protecting groups such as Dmab (which is incompatible with loading conditions for indolyl resin), the mild deprotection conditions (AcOH/MeOH/TIS, 80:15:5) minimized side reactions, allowing selective cleavage of the Trt group. Macrocyclization was effected by HCTU, a uronium-based coupling reagent that enabled robust cyclization under mild conditions while maintaining compatibility with the solid-phase setup. Following synthesis, cleavage was performed using TFA/water (95:5) alone, as Trt protecting groups had already been removed in a prior step. This eliminated the need for scavengers like TIS, which would have been challenging to remove since the synthesized macrocycles are incompatible with ether precipitation. This strategy enabled introduction of diverse P1′ residues and a phenylstatine transition-state mimic at the P1 position, yielding a focused combinatorial library of 624 unique macrocycles.

We constructed the library to investigate the impact of aliphatic linker length on May1 inhibition as well as cyclization efficiency. The purity of all compounds in the library was within 20–80% range (Figure S2), assayed using LC–MS with evaporative light scattering detection used for quantitation. The C11 linker yielded the highest cyclization efficiency, and assemblies with linkers longer than C6 also routinely exhibited purities above 50%. In contrast, shorter linkers introduced steric constraints that hindered amide bond formation, leading to a sharp drop in purity, which was below 20% on average for C4 linker. Notably, no products were observed for C2 and C3 linkers, likely due to excessive strain preventing successful cyclization in this setup.

### Inhibitor Motif Identification Using a Combinatorial Approach

**Figure 1:**
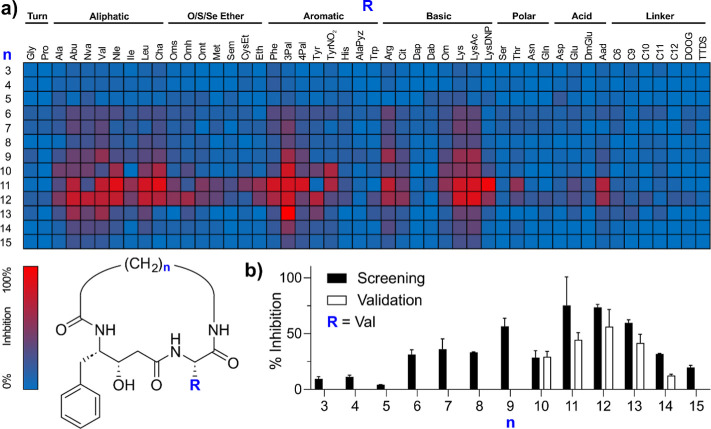
Inhibitor potency dependence on P1′ residue and linker length. (a) Screening of the focused combinatorial macrocycle library conducted at 5 nM final concentration. (b) Effect of macrocycle linker length on inhibition with P1′ valine. Activity at 5 nM was calculated from determined *K*_*i*_ values for macrocycles with *n* = 10–14.

To identify optimal structural motifs for May1 inhibition, we screened the focused library to evaluate the influence of linker lengths and P1′ residues on inhibitory potency. The library design allowed for exploration of a diverse chemical space, and our initial high-throughput screening revealed that inhibitory activity is strongly influenced by both the linker length and the identity of the P1′ residue (Figure 1a).

Optimal activity was observed with linkers C11–C14, which also provided the highest yields of macrocyclization (Figure S2). Among P1′ residues, aromatic substituents, such as 3-pyridylalanine (3Pal), were particularly effective in enhancing potency, possibly due to their ability to form stabilizing hydrophobic interactions within the enzyme’s active site given that aliphatic residues with specific branching (e.g., Val and Leu, but not Ile) are also well-tolerated. Aliphatic and basic residues were also tolerated and showed good activity, although they were slightly less effective than their aromatic counterparts.

To validate and refine these findings, a focused series of purified inhibitors was synthesized and tested to confirm the structure–activity relationships. This approach revealed that macrocycles containing a C12 linker and an aromatic P1′ residue consistently exhibited low nanomolar inhibitory activity, with the best-performing compound (**7**) showing *K*_*i*_ of 3.1 ± 0.4 nM (Table 1a). While aromatic P1′ residues provided robust inhibition, lysine substitutions proved to be viable alternatives for further diversification as evidenced by the strong performance of the acetyl and 2,4-dinitrophenyl lysine derivatives in the initial screening. This activity was further enhanced by modifying lysine with methylcarbamate, which yielded **9** with *K*_*i*_ of 400 ± 200 pM.

**Table 1:**
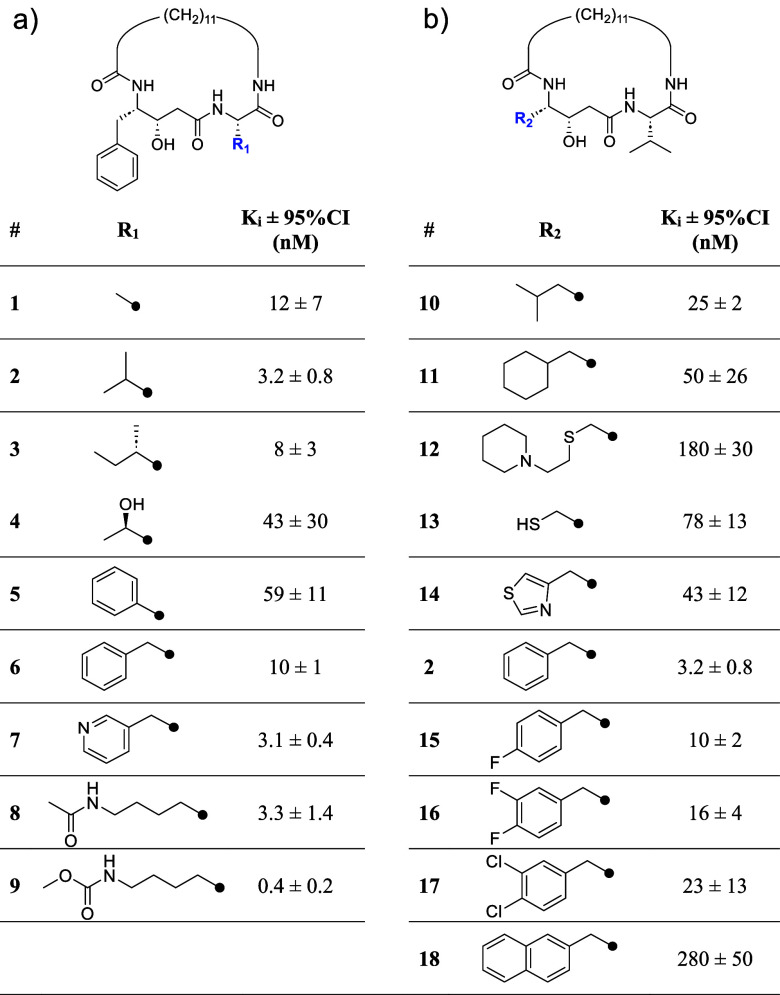
SAR of Selected N–C Cyclized Inhibitors with a C12 (*n* = 11) Bridge Varying in Statine and P1′ Residues

a Effect of P1′ residue on activity.

b Comparison of different statine analogues.

Expanding on our previous work,^11,17^ we explored the effects of various statine-based transition state mimics in P1. For this series of compounds, we selected Val as the P1′ residue due to its robust inhibitory activity and broad P1 compatibility. Lysine methylcarbamate, despite its higher potency exemplified in **9**, posed challenges for incorporation into this series due to its variable stability under the deprotection conditions used in synthesis. May1 showed a strong preference for aromatic P1 residues, with additional steric hindrance beyond phenyl being disfavored (Table 1b). While the changes in substitution of statine side chain did not yield any improvement in activity, there was a more than 10-fold difference in inhibitory activity between phenylstatine-bearing inhibitor **2** and its cyclohexyl analogue **11**, suggesting a possible role for π–π interactions alongside hydrophobic effects in stabilizing binding.

The influence of chain length on inhibitory activity was further explored within the preferred region of the initial combinatorial screening range (C11–C15) on a focused series of purified inhibitors. By fixing the P1′ residue to valine and extending the aliphatic linker length to C15, we identified a distinct minimum at C13, with **20** achieving a *K*_*i*_ of 2.0 ± 1.2 nM (Table 2). Moreover, inhibitory activity of P1′ valine-containing macrocycles followed closely the results of initial screening (Figure 1b).

**Table 2:**
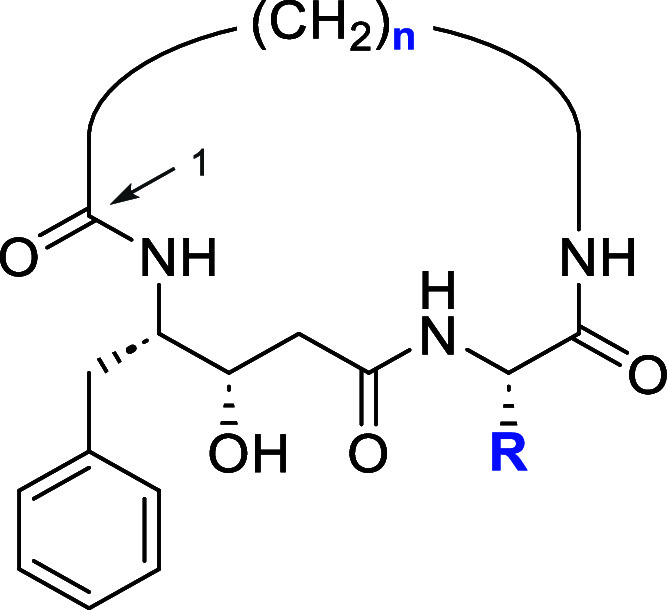

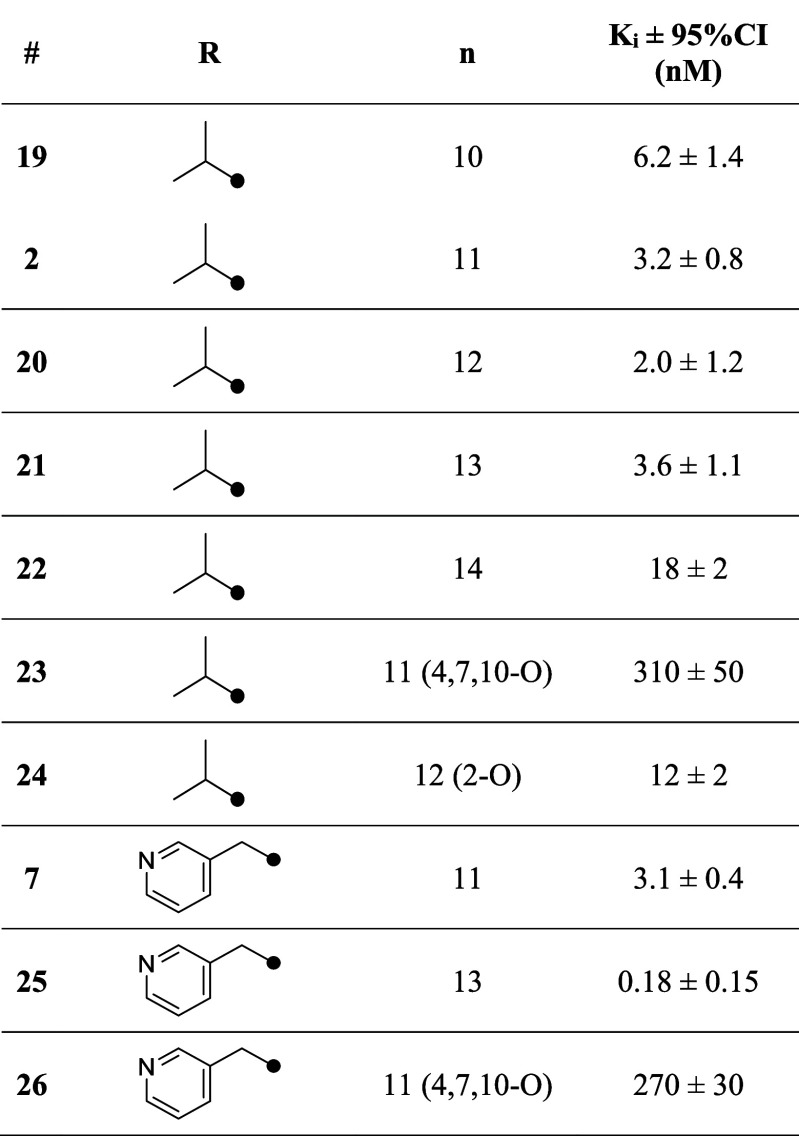
Optimization of Linker Chain Length for Phenylstatine-Based Inhibitors Bearing 3-Pyridylalanine or Valine Residues in P1′^a^

a Positions of oxygens in **23**, **24**, and **26** are indicated with respect to the carbonyl carbon highlighted by arrow.

Although modifications to the linker, such as the introduction of ether groups, were explored to enhance solubility and promote additional polar interactions, they were ultimately disfavored, likely due to the disruption of hydrophobic interactions critical for binding. Building on these findings, we examined whether integrating 3Pal, the most favorable P1′ residue identified in the combinatorial screening, with an extended aliphatic linker could further enhance inhibitory potency. This led to the development of **25**, which demonstrated a significantly improved *K*_*i*_ of 180 ± 150 pM, aligning with the activity trends observed in the combinatorial screening.

### Lead Macrocyclic Inhibitor Forms Key Interactions with the May1 Active Site

**Figure 2:**
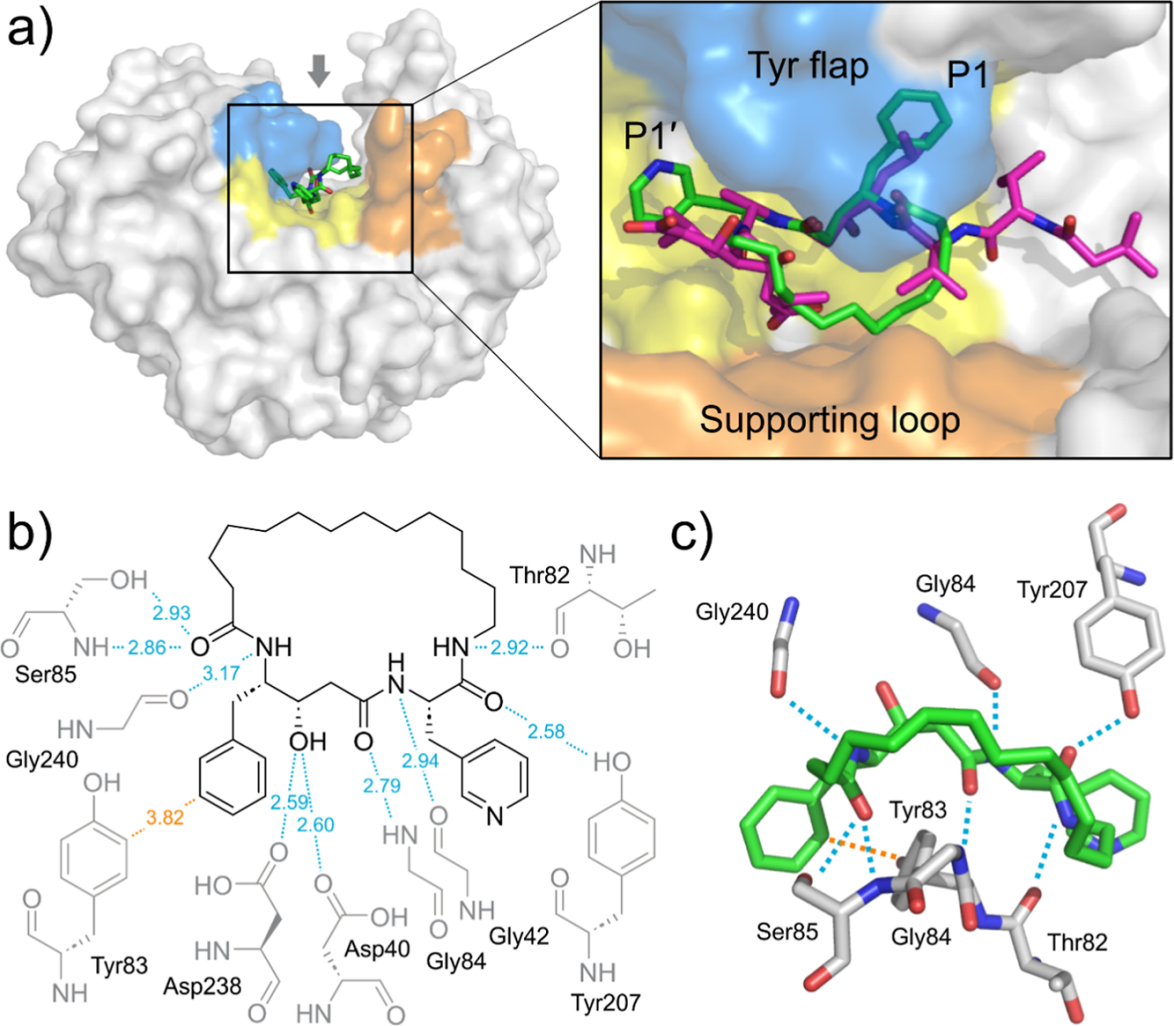
Structural overview of the effect of macrocyclization on binding mode. (a) Crystal structure of recombinant May1 in complex with **25** superimposed with PepA (PDB 6R61/6R6A). (b) Ligand–protein interaction diagram of the May1–**25** complex. (c) Ligand–protein interacting residues location. Perpendicular π–π interaction of the phenylstatine core with Tyr83 is shown with orange dotted line. Hydrophobic interactions (Ile38, Ser43, Ile81, Leu119, Leu127, Ile135, Thr241, Met316, Met321, Ile330) are excluded for clarity.

Next, we crystallized May1 (recombinant protein spanning residues 17–434 with a C-terminal Avi tag^17^) in complex with macrocyclic inhibitor **25** (Figure 2a). The structure was determined by molecular replacement using the structure of free May1 (PDB code 6R5H) as a model. The complex crystallized in the orthorhombic *C*222_1_ space group with one molecule in the asymmetric unit, and the structure was refined to a resolution of 1.81 Å (Table S1) with a well-defined continuous electron density map for the bound ligand and active site.

We did not observe any significant global differences between the May1–**25** structure and the structures of free May1 and May1 complexed with the aspartyl protease inhibitor pepstatin A (PepA). The root-mean-square deviation (RMSD) for the superposition of protein backbones was 0.156 Å for May1–**25** versus free May1 (6R5H) and 0.114 Å versus May1-PepA (6R6A), consistent with the 0.129 Å difference between the two reference structures. As observed previously,^17^ the structure contained a glycosylated asparagine at position Asn187, modeled as GlcNAc-β(1 → 4)GlcNAc-β-Asn187.

PepA adopts an extended conformation within the S4–S2′ substrate-binding groove of May1. In contrast, **25** primarily targets the central active site (S1 and S1′ subsites), leaving peripheral subsites unoccupied and inducing a slightly more open active site conformation relative to the PepA complex. Despite its smaller size and reduced interaction surface, **25** retains essential inhibitory contacts within the catalytic pocket (Figure 2b). These include critical hydrogen bonds with the catalytic aspartic residues (Asp40 and Asp238) via its central functional groups, effectively mimicking the transition-state interactions formed by PepA.

A notable feature of the May1–**25** complex is the perpendicular π–π interaction between the phenylstatine residue and Tyr83 (Figure 2c). This interaction occurs with a near-canonical distance of 3.82 ± 0.19 Å between the apical phenylstatine carbon and the Tyr83 aromatic carbons, consistent with previously characterized perpendicular π–π interactions.^19^ This highlights the enhanced hydrophobic stabilization introduced by the phenylstatine moiety, further compensating for the reduced size of **25** compared to PepA. While the P1′ residue is also in the vicinity of Tyr83 (Figure 2c), no comparable interaction is observed with the 3-pyridyl residue, which is positioned farther away at 5.85 ± 0.55 Å, ruling out any significant aromatic binding contribution from this site. Moreover, the closest possible partners for hydrogen bonding of the heterocyclic nitrogen—Thr82 Nα at 4.77 Å or Tyr207 Oη at 4.20 Å—are too far to engage meaningfully. It therefore remains unclear what structural advantage the 3-pyridyl residue has over phenyl residue that would translate into the observed improvement of inhibitory activity.

Additionally, **25** establishes extensive van der Waals interactions with May1, facilitated by the optimized inhibitor core and macrocyclic strain. The macrocyclic strain in **25** plays a critical role in aligning the central functional groups with the catalytic dyad and adjacent active site residues, promoting a preorganized binding conformation. This preorganization reduces entropic penalties upon binding and may result in the tighter binding observed within the active site, further enhancing inhibitor potency despite the smaller interaction surface.

### Computational Analysis of the Binding Mode of Compound **25**

We employed semiempirical quantum mechanical (SQM)-based scoring with ligand fragmentation to assess the binding free energy contributions of different inhibitor parts and to gauge their importance in May1–**25** binding. The first step was to determine the location of the proton on the catalytic aspartic dyad side chains in the protein–ligand complex. We modeled two possible positions of the proton on OD1 of Asp40 and on OD2 of Asp238. At the semiempirical level, the total stabilization energy indicated that the protonated Asp238 variant was significantly more stable (∼18 kcal/mol) than the protonated Asp40 variant (Table S2). This protonation state was therefore used in all subsequent calculations.

Using the model with the protonated OD2 of Asp238, we next employed SQM2.20 scoring function^20^ to determine the total interaction “free” energy (comprising gas-phase energy and solvation free energy components) of **25** bound to May1. Additionally, we compared the binding affinity of **25** with that of **21**, where the P1′ 3-pyridylalanine was substituted with Val. The interaction “free” energy in solvent and the total SQM2.20 score identified **25** as the more potent inhibitor, with improvements of 5.2 and 8.3 kcal/mol, respectively, over **21** (Table S3). This aligns with the experimentally determined *K*_*i*_ values (0.18 ± 0.15 nM for **25** vs 3.6 ± 1.1 nM for **21**), validating our model and confirming the reliability of the SQM-based scoring approach in capturing key determinants of inhibitor binding.

**Figure 3:**
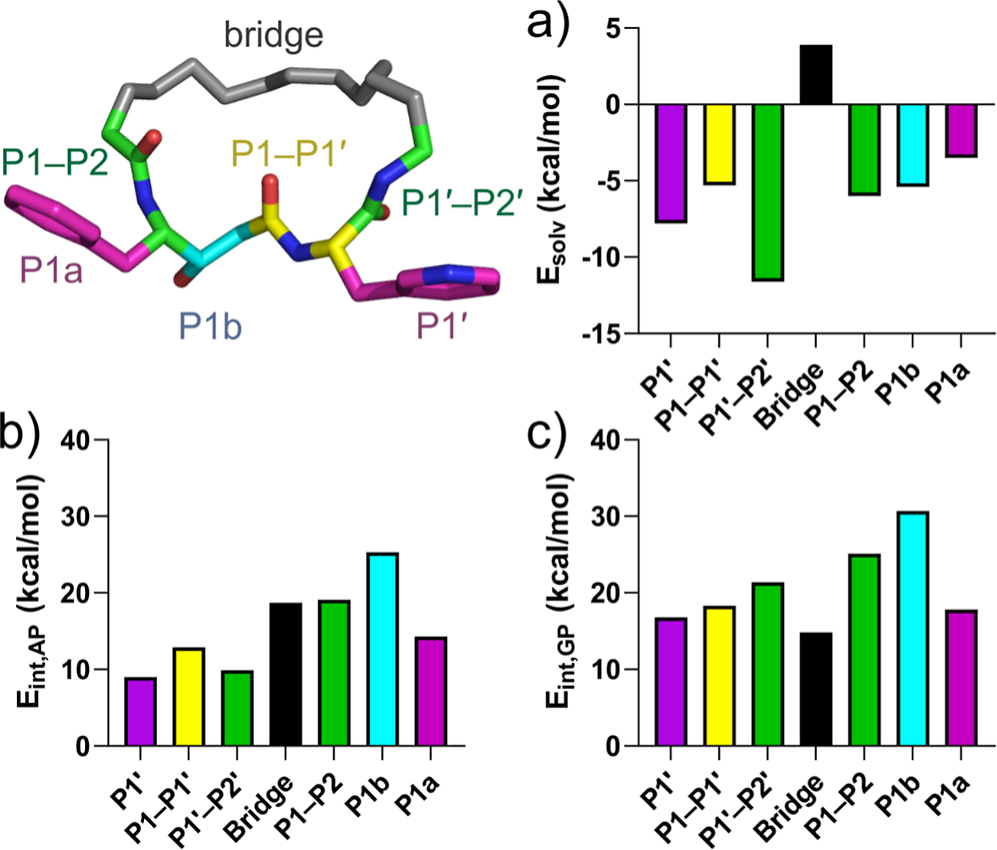
Quantum chemical calculations of interaction “free” energy contributions of individual parts of compound **25** in the May1 active site. The 3D view illustrates the fragmentation of the inhibitor into color-coded segments. (a) Solvation penalty contribution of individual inhibitor parts. (b) Interaction “free” energy contributions. (c) Interaction energies in the gas phase.

Fragment-based analysis provided insight into the energetic contributions of different ligand regions to binding (Figure 3). The total interaction ‘free’ energy contribution of compound **25** was 75.9 kcal/mol, with individual fragments contributing to varying extents (Figure 3 and Tables S4 and S5). The statine central fragment (P1b, 25.3 kcal/mol, Figure 3b) acting as the key transition state mimic and the central peptide bond in P1–P1′ fragment (12.9 kcal/mol) had substantial stabilizing effects with similarly high desolvation penalty contributions of ∼5 kcal/mol (Figure 3a). The second peptide bond (P1′–P2′ fragment) contributed 9.9 kcal/mol, with the strongest relative desolvation penalty contribution (11.6 kcal/mol). The bridge region had a higher stabilizing effect in solvent (18.7 kcal/mol), than in vacuo (14.8 kcal/mol, Figure 3c), thus decreasing total desolvation penalty of **25**. The third peptide bond in P1–P2 segment provided one of the highest binding contributions (19.1 kcal/mol) with a low desolvation penalty of 6 kcal/mol, underscoring its importance in stabilizing the complex.

The results highlight the critical role of the P1 phenyl group (P1a fragment) in enhancing binding affinity by π–π interactions (contributing by 14.3 kcal/mol with the second lowest desolvation penalty of 3.5 kcal/mol), while also showing that modifications of the P1′ region, where the 3-pyridyl group interacts with structurally significant water Wat175, as well as Ile81 and Ile135, might further improve potency, as it brings the smallest contribution (9.0 kcal/mol) to the total binding while having the second highest desolvation penalty of 7.8 kcal/mol. These insights thus demonstrate the utility of fragmentation-based scoring in guiding the rational design of improved inhibitors by identifying key structural elements critical for binding.

### Lead Compounds Display Low Off-Target Activity

To evaluate the feasibility of progressing to in vivo experiments, we assessed the in vitro off-target activity and cytotoxicity profiles of the lead compounds **9** and **25**. Off-target activity, particularly against host aspartyl proteases, remains a major concern in the design of protease inhibitors. Both compounds demonstrated high selectivity for May1 and limited activity against several key human aspartyl proteases (Table 3). Importantly, **25** displayed low off-target effects against renin (*K*_*i*_ > 500 μM) and pepsin (*K*_*i*_ = 1.9 ± 0.9 μM), while moderate inhibition was observed for cathepsin D (*K*_*i*_ = 37 ± 3 nM) and cathepsin E (*K*_*i*_ = 91 ± 10 nM). This profile was similar to that of **9**, albeit with an inverted selectivity against cathepsin D (*K*_*i*_ = 64 ± 8 nM) and cathepsin E (*K*_*i*_ = 26 ± 2 nM).

**Table 3:** In Vitro Off-Target Profiles of Lead Compounds

compound	*K*_*i*_ ± 95% CI					
	May1 (pM)	HIV-1 Pr (μM)	pepsin (μM)	renin (μM)	cathepsin D (nM)	cathepsin E (nM)
**9**	400 ± 200	>500	7.6 ± 2.5	>100	64 ± 8	26 ± 2
**25**	180 ± 150	>500	1.9 ± 0.9	>500	37 ± 3	91 ± 10

To assess the overall safety profile of the compounds, cytotoxicity was evaluated in various mammalian cell lines, including CEM, HL60, MCF-7, and HeLa (Table 4). Compound **9** exhibited very low cytotoxicity with CC_50_ > 100 μM across all tested cell lines. Compound **25** showed moderate CC_50_ values ranging from 9.9 ± 1.2 μM (CEM) to 34.1 ± 0.5 μM (HeLa), which was considered acceptable for further development.

**Table 4:** Cytotoxicity Profiles of Lead Compounds

compound	CC50 ± s.d.			
	CEM (μM)	HL60 (μM)	MCF-7 (μM)	HeLa (μM)
**9**	>100	>100	>100	>100
**25**	9.9 ± 1.2	14.4 ± 0.9	19.3 ± 1.5	34.1 ± 0.5

While the cytotoxicity levels of **25** warrants cautious interpretation, they remain within a range of clinically used drugs such as antivirals nelfinavir and saquinavir, which exhibit CC_50_ across multiple cell lines in the ranges of 5–19 and 7–32 μM, respectively.^21^ This, combined with their favorable off-target activity profiles, suggests that both compounds are promising candidates for in vivo evaluation.

### Pharmacokinetic Properties of Hit Compounds

Both **25** and **9** exhibited good plasma stability, with no significant degradation observed over 120 min in human or mouse plasma (Table S6). Compound **25** demonstrated a moderate microsomal clearance profile, with half-lives of 44 ± 4 and 35 ± 8 min in human and mouse liver microsomes, respectively (Table S7). In contrast, **9** displayed high stability in liver microsomes from both species, with no observable degradation after 45 min.

Caco-2 permeability assays revealed a substantial basolateral-to-apical efflux for both compounds (Table S8), indicative of potential challenges with intestinal absorption and transport. Efflux ratios for **25** and **9** were 38 and 25, respectively, with **25** showing lower permeability (*P*_app_ = 0.9 × 10^–6^ cm/s) compared to **9** (*P*_app_ = 2.9 × 10^–6^ cm/s) in the apical-to-basolateral direction. These results suggest the potential involvement of efflux transporters in limiting oral bioavailability.

In vivo pharmacokinetic tests revealed modest but consistent oral bioavailability for both **25** (12.5%) and **9** (6.0%). Intravenous administration revealed significant differences in clearance rates (Table S9), with **25** exhibiting faster clearance (*T*_1/2_ = 6.9 min) than **9** (*T*_1/2_ = 79.6 min). However, upon oral gavage, **25** had a longer half-life (*T*_1/2_ = 83.7 min) than **9** (*T*_1/2_ = 42.4 min). Despite the faster clearance, **9** achieved more than 2-fold higher plasma concentrations after oral administration (*C*_max_ = 21.2 ng/mL or 37.0 nM) compared to **25** (*C*_max_ = 10.4 ng/mL or 18.5 nM).

These findings suggest that both compounds may require frequent dosing or alternative formulations. Therapeutic levels could be realistically achieved in mouse models with administration in drinking water or via osmotic pump, as peak concentrations for both compounds exceed the *K*_*i*_ values of respective compounds by more than 2 orders of magnitude.

Interestingly, while **9** had better adsorption kinetics, it was associated with significant morbidity upon i.v. administration. Specifically, 3 out of 6 mice treated with **9** required euthanasia before the study end point, potentially limiting its utility for further development. In contrast, **25** did not exhibit such adverse effects, supporting its selection for continued evaluation.

### Antifungal Activity of May1 Inhibitors in *C. neoformans* Yeast Culture

To evaluate the antifungal efficacy of the May1 inhibitors, we tested their activities against cultured *C. neoformans* yeast. May1 activity is dispensable during logarithmic-phase growth in yeast nitrogen base (YNB) minimal medium but is required during stationary phase, after the medium has acidified.^15^ Specific May1 inhibition therefore results in reduced end-point culture densities that can be rescued by buffering the medium to prevent acidification.

**Figure 4:**
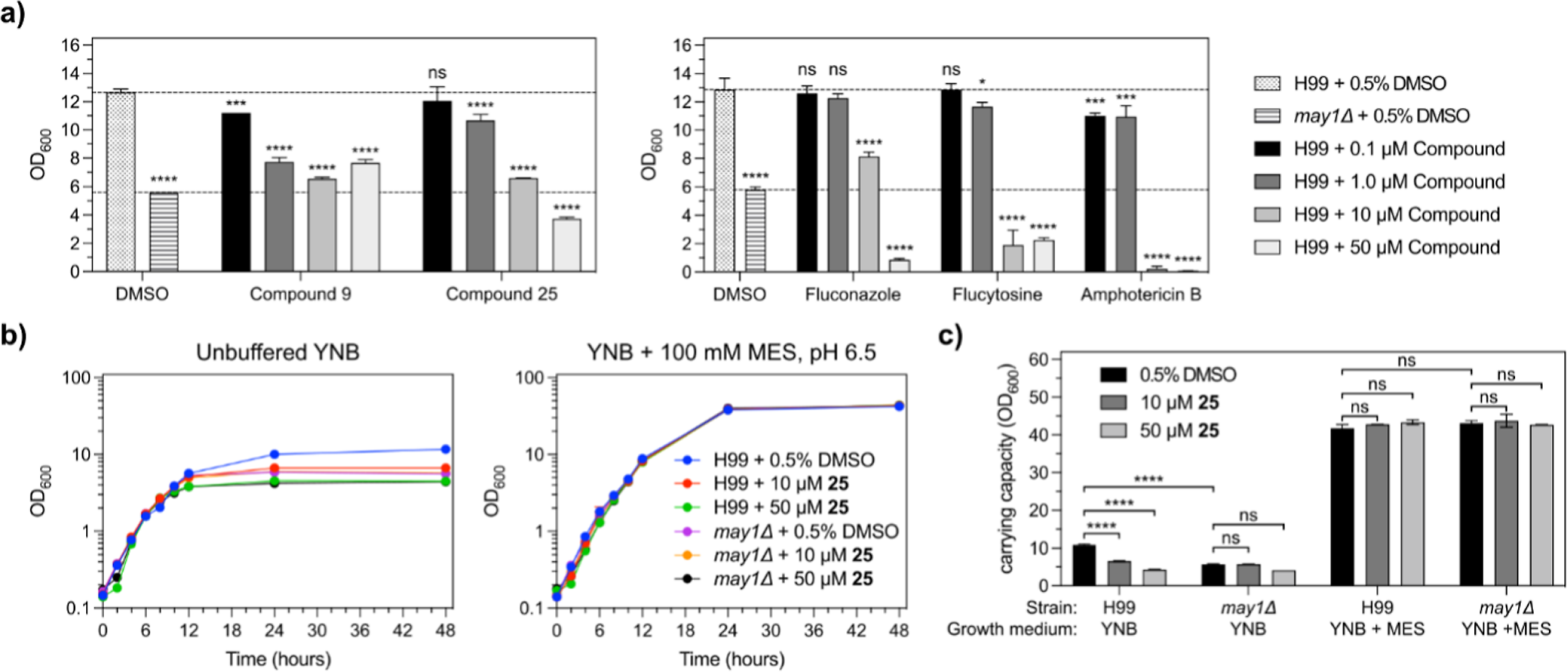
Evaluation of May1 inhibitor activity against cultured *C. neoformans* yeast. Error bars represent standard deviation of the mean from triplicate cultures. (a) End-point saturation densities (OD_600_) of yeast cultured for 48 h in unbuffered YNB minimal medium containing May1 inhibitors **9** and **25** (left), and three selected clinically used antifungals (right). Dashed lines represent end-point optical densities of the 0.5% DMSO-treated H99 and *may1*Δ controls. Statistical analysis was performed using one-way ANOVA with Dunnett’s multiple comparisons tests versus the H99 + 0.5% DMSO control: ns, not significant, *p < 0.05, **p < 0.01, ***p < 0.001, ****p < 0.0001. (b) Growth curves of H99 and *may1*Δ yeast treated with compound **25** or a 0.5% DMSO vehicle control in either unbuffered YNB medium (left) or YNB buffered to pH 6.5 (right). (c) Carrying capacities for growth curves in (b) determined using Growthcurver.^22^ Statistical analysis was performed using one-way ANOVA with Šídák’s multiple comparisons tests: ns, not significant, *p < 0.05, **p < 0.01, ***p < 0.001, ****p < 0.0001.

*C. neoformans* wild-type (H99) and *may1Δ* mutant yeast strains were cultured in YNB minimal medium supplemented with **9** and **25** at concentrations ranging from 0.1 to 50 μM (Figure 4a, left). Consistent with previous observations, the *may1Δ* mutant strain achieved significantly lower end-point densities compared to the H99 wild-type strain, mimicking the phenotype associated with May1 deficiency.^15^ Treatment of H99 yeast with **9** and **25** led to a dose-dependent reduction in end-point densities, with both compounds at 10 μM nearly phenocopying the *may1Δ* mutant phenotype. To provide a pharmacologically relevant context for interpreting these results, we also analyzed the effect of clinically used antifungals (Figure 4a, right). Under the same growth conditions, fluconazole, flucytosine, and amphotericin B also reduced end-point density, with amphotericin B causing near-complete abrogation of growth at 10–50 μM. While these reference drugs showed stronger growth inhibition than compounds **9** and **25** in this assay, the May1 inhibitors still approached the *may1Δ* phenotype at similar concentrations, suggesting meaningful on-target activity despite their distinct mechanism of action.

Notably, treatment with **25** at a concentration of 50 μM resulted in an end-point density suppression that exceeded the reduction observed in the *may1Δ* control, a trend that was similarly reflected in the growth curve analysis in unbuffered medium (Figure 4b). The growth curves were statistically analyzed for carrying capacity^22^ which showed a significant decline for the wild-type H99 strain at both 10 and 50 μM concentration of **25** (Figure 4c). To assess the specificity of **25** for May1, we also examined the growth in medium buffered to pH 6.5, which negates the need for May1 activity and allows for higher end-point densities by minimizing the effects of media acidification. Under these conditions, treatment with **25** did not significantly impact the growth of either the wild-type or the *may1Δ* mutant, even at 50 μM (Figure 4c).

## Discussion

In this study, we developed potent macrocyclic inhibitors targeting *C. neoformans* May1. A notable advantage of our approach is the ability to synthesize a high number of inhibitors rapidly in a 384-well plate format. This high-throughput capability allows for efficient exploration of diverse chemical spaces and supports the identification of optimal linkers and residue combinations. While the parallel synthesis approach does not always yield individual compounds with sufficient purity to resolve detailed SAR, it remains a powerful tool for rapidly surveying a large chemical space. The purity of all compounds in the library was within 20–80% range (Figure S2), underscoring the variability inherent to high-throughput parallel synthesis. However, with appropriate validation experiments, this approach enables the identification of promising scaffolds that can be further refined through focused synthesis and rigorous biochemical evaluation.

SAR analysis identified several pivotal factors influencing inhibitor activity with C11–C14 aliphatic linkers combined with a phenylstatine core at the P1 position exhibiting the strongest inhibitory activity. While May1 does not appear to tolerate mixed ether linkers within this length range due to the hydrophobic nature of its binding pocket, these linkers could be utilized for other targets to enhance solubility and promote additional polar interactions, broadening the applicability of the macrocyclization platform. Modifications at the P1′ position further highlighted the role of aromatic residues, notably 3-pyridylalanine, in enhancing binding affinity. Presenting another hydrogen bond acceptor, 3-pyridylalanine-containing macrocycles also showed improved water solubility while also exhibiting a 3-fold activity improvement in comparison to Phe-containing inhibitors.

Notably, charged residues were tolerated only upon significant extension of the charge-bearing side chain to 4 carbons, an effect which was consistent regardless of polarity as can be seen on the examples of inhibitors containing P1′ Lys and α-aminoadipoyl residues. Moreover, masking the charge while maintaining a hydrogen bond donor leads to improvement in inhibition, which is exemplified with derivatization of Lys with acetyl (**8**) or methylcarbamate (**9**).

Our previous study on linear inhibitors of May1 identified phenylstatine-based scaffolds as potent transition-state mimics, effectively inhibiting the enzyme with nanomolar affinities.^17^ However, these inhibitors exhibited a higher number of rotatable bonds and hydrogen bond donors and acceptors, which likely contributed to their inferior binding characteristics and precluded their use in animal models. In contrast, the macrocyclic inhibitors described in this study benefit from restricted conformational flexibility, reducing the entropic cost of binding and improving target engagement. This effect is reflected in the nearly 2 orders of magnitude improvement in potency, with the best macrocyclic inhibitor, compound **25**, achieving a *K*_*i*_ of 180 pM compared to 12 nM for the best linear inhibitor Z-Pst-Glu-Hph-NH_2_. These findings highlight how macrocyclization can enhance inhibitor potency by optimizing spatial preorganization while preserving the key interactions critical for enzymatic inhibition.

Structural analysis of the May1–**25** complex revealed a unique binding mode in which **25** primarily engages the S1 and S1′ subsites, leaving peripheral regions unoccupied. S1 subsite also disfavors residues larger than phenyl which contrasts with cathepsin D for which naphthyl residue was the most favorable in P1 position.^11^ Moreover, there was a more than 10-fold difference in inhibitory activity between phenylstatine-bearing inhibitor **2** and its cyclohexyl analogue **11** and this effect less than 2-fold with cathepsin D.^11^ This can be explained by the improper orientation of residues Phe126/131^11^ which cannot participate in perpendicular π–π interaction like Tyr83 of May1. The linker length optimum for cathepsin D was shown to be C12–C13,^11^ however compound **25** with C14 linker showed *K*_*i*_ of 37 ± 3 nM and compound **9** with C12 showed *K*_*i*_ of 64 ± 8 nM. While unexpected, this observation mirrors the inhibitors’ activity against May1 where the optimal linker length for 3-pyridylalanine was shown to be C14 whereas for Val the optimum is C13. This highlights the need to conduct screening bidimensionally instead of isolating the linker length and residues as independent variables. Moreover, both compounds **9** and **25** showed no appreciable inhibition of HIV protease with *K*_*i*_ of more than 500 μM. This is despite the preference for P1′ Phe and Gln in HIV protease inhibitors^23^ and indicates that macrocyclization may preclude inhibition of HIV protease due to steric constraints.

Among the inhibitors tested, **25** emerged as the lead compound, exhibiting a *K*_*i*_ of 180 ± 150 pM against May1 and demonstrating strong efficacy in antifungal assays. The antifungal activity of **25** was confirmed in *C. neoformans* yeast cultures, in which the compound exhibited dose-dependent reductions in end-point saturation densities, and closely phenocopying the May1-deficient strain at 10 μM concentration. At 50 μM, however, treatment with **25** led to an even greater reduction in end-point density than the may1Δ control (Figure 4a, left). This enhanced suppression at high concentrations suggests that **25** may exert additional effects beyond May1 inhibition at high concentrations. Another explanation could be that, unlike genetic deletion, which allows cells to adapt by activation of compensatory metabolic pathways to the absence of May1 during the ongoing culturing, immediate pharmacological inhibition may impose a more severe metabolic burden, leading to an exaggerated growth defect. This explanation is supported by the fact that growth of wild-type and may1Δ mutant in medium buffered to pH 6.5, which negates the need for May1 activity and allows for higher end-point densities due to diminished effects of media acidification,^15^ is not significantly affected by the treatment with **25** even at 50 μM (Figure 4c).

To provide a pharmacologically relevant context for interpreting these results, we also analyzed the effects of three clinically used antifungals—fluconazole, flucytosine, and amphotericin B—under the same assay conditions (Figure 4a, right). All three drugs reduced end-point density more than **9** and **25** at concentrations of 10–50 μM. While compound **25** does not match the potency of these established antifungals, its selective targeting of a previously unexploited protease offers a distinct and mechanistically novel approach. This is particularly relevant given that current antifungal agents are often associated with significant limitations, including toxicity, drug interactions, and the emergence of resistance.^24^

Moreover, recent findings indicate that May1 plays a role beyond proteolytic activity by remodeling the cryptococcal cell wall in acidic conditions, specifically by reducing chitosan levels through degradation of the primary chitin synthase Chs3.^25^ Given that May1 inhibition could prevent this chitosan loss, compound **25** could be a valuable tool to further dissect the role of May1 in cell wall maintenance. Future studies could explore whether this inhibitor impacts fungal cell wall integrity or host immune recognition, particularly in vivo where chitosan reduction may be critical for fungal adaptation.^26^ In addition to its potential as a research tool, **25** demonstrated favorable drug-like properties comparable to those reported for other medicinally relevant macrocycles,^3,10,13^ such as minimal off-target activity against human proteases and cytotoxicity levels similar to clinically used drugs such as the antivirals nelfinavir and saquinavir.^21^

These observations were also confirmed by in vivo mouse experiments which showed that compound **25** is well tolerated and orally bioavailable. In contrast, compound **9** showed even lower cytotoxicity with CC_50_ values >100 μM but was poorly tolerated in vivo in identical dosage. This combination of high selectivity, low toxicity, and potent antifungal activity positions **25** as a promising candidate for further development. The macrocyclization strategy effectively improved the pharmacological properties of the inhibitors, offering enhanced stability and target selectivity compared to linear analogs, while improving May1 inhibitory potency by almost 2 orders of magnitude.^17^ These findings underscore the importance of linker length and core optimization in achieving such high potency. Nonetheless, further optimization will be required to improve pharmacokinetic properties and to address key challenges such as central nervous system exposure, as effective treatment of cryptococcal meningitis ultimately demands compounds capable of crossing the blood–brain barrier.

## Conclusion

We developed a series of macrocyclic inhibitors targeting *C. neoformans* May1, utilizing an innovative on-resin macrocyclization strategy. The lead compound, **25**, demonstrated subnanomolar potency against May1, strong antifungal activity in yeast culture, acceptable levels of cytotoxicity in mammalian cells and oral bioavailability in mouse model. Structural analysis revealed that macrocyclization not only enhances target selectivity but also promotes tighter binding through optimized alignment and hydrophobic stabilization. These findings establish **25** as a valuable stepping stone toward the development of novel antifungal therapies and provide a template for future optimization efforts aimed at addressing the urgent need for effective treatments against cryptococcosis.

## Methods

### Macrocyclic Library Testing

Activity assays were performed in 384-well plates with transparent flat bottoms (Greiner 781097) in a stop-point format using a modified version of a previously described procedure.^17^ Assay mixtures consisted of 15 μL of 50 mM sodium acetate, pH 5.0, 50 mM sodium chloride, 0.05% Tween-20 (Buffer R) with 33 μM IQ-2 substrate [AMC-GSPAFLAK(DNP)dR-NH_2_]. Test compounds were dispensed by a LabCyte Echo 550 acoustic dispensing system in triplicate with a final DMSO content of 0.01% (v/v) and a final inhibitor concentration of 5 nM. Reactions conducted at 37 °C were started by sequential addition of May1(17-434)-Avi^17^ in 10 μL Buffer R to each well by an EnSpire Multilabel Reader to a final enzyme concentration of 0.98 nM and stopped after 20 min by addition of 12 μL of 50 μM pepstatin A in Buffer R. Reaction fluorescence (excitation at 328 nm, emission at 393 nm) was analyzed using a plate reader (Tecan Infinite M1000). Each plate contained 12 positive controls with 1 μM pepstatin A and negative controls with DMSO to determine *Z*′ = 0.820 ± 0.046 (*R* = 0.899–0.783). Reported fractional inhibition values for a given inhibitor were calculated using its median reaction fluorescence value *F*_i_ as , where *F*_+_ and *F*_–_ are the median fluorescence values of positive and negative control wells, respectively.

### *K*_*i*_ Determination

The library screening assay was adjusted to contain a triplicate of a 3-fold dilution series of inhibitors with ten points total designed to be centered around the expected *K*_*i*_ estimated from preliminary assays and final DMSO concentration normalized to 0.16% (v/v). *K*_*i*_ values were determined by nonlinear regression analysis with the Morrison equation (GraphPad Software GraphPad Prism 6) using *K*_m_ value of IQ-2 substrate (21 ± 4 μM), substrate concentration *c*_s_ = 20 μM and enzyme concentration 5 nM. Inhibition curves are included in Figure S3. For the purposes of screening validation, the fractional inhibition  at concentration *c*_a_ = 5 nM was calculated from *K*_*i*_ values as .

### Assays for Off-Target Activity

Selected inhibitors were screened for off-target activity against porcine pepsin (Sigma-Aldrich P7012), human renin (Biovision 6300), human cathepsin D (Athens Research & Technology 16-12-030104), and human cathepsin E (Biovision 7842). Established protocols were used with the substrates H-R-E(EDANS)-IHPFHLVIHT-K(DABCYL)-R-OH^27^ (*K*_m_ = 2.5 μM, Anaspec AS-62022) for renin, BSA-bromophenol blue^28^ (*K*_m_ = 80 μM) for pepsin, and ACC-GKPILFFRLK(DNP)-(dR)-NH_2_^29^ (*K*_m_, CatD = 3.7 μM, *K*_m_, CatE = 3.3 μM, Anaspec AS-61793) for cathepsins D and E. Inhibition curves are included in Figure S4.

### Yeast Culture Activity Assay

*C. neoformans* strains H99 (CM018) and *may1Δ* (CM1383)^15^ were cultured in YNB medium containing 2% glucose. To assess May1 inhibitors for reduction of saturation culture density, overnight cultures were diluted to OD600s of 0.1 in the presence of 0.5% DMSO vehicle or 0.1, 1.0, 10, or 50 μM compound. Cultures were incubated at 30 °C on a tube rotator, and OD600s were measured after 48 h. As controls, yeast were similarly treated with the traditional antifungal drugs fluconazole (Santa Cruz sc-205698), flucytosine (TCI F0321), or amphotericin B (Sigma A4888). For full growth curves, logarithmically growing cultures were pelleted and resuspended to OD600s of 0.1 in either unbuffered YNB or YNB containing 100 mM MES, pH 6.5. Cultures were treated with 10 or 50 μM compound **25**, or 0.5% DMSO vehicle and were incubated at 30 °C on a tube rotator. OD600 values were measured at 2-h intervals for 12 h and after 24 and 48 h of growth. Carrying capacities of growth curves were determined using the Growthcurver^22^ package version 0.3.1 implemented in RStudio version 2024.12.1 + 563 running R version 4.4.3. Statistical analyses are indicated in figure legends and were performed in GraphPad Prism version 10.2.3.

### Crystallization and X-ray Data Collection

Diffraction-quality cocrystals of May1 and **25** were obtained at 25 °C using the hanging-drop vapor diffusion technique previously described^17^ with some modifications. The May1-inhibitor complex was formed by incubating May1(17-434)-Avi protein (1 mg/mL) in Buffer P with **25** (100 μM final concentration) at 4 °C for 1 h. The complex was then concentrated, and excess inhibitor was removed by ultrafiltration using an Amicon 3K MWCO centrifugal device.

Cocrystals were then obtained by mixing equal volumes of concentrated complex (at approximately 80 mg/mL) with reservoir solution composed of 200 mM lithium sulfate, 45% (v/v) PEG-400, 100 mM sodium acetate, pH 4.5. Drops were streak-seeded with previously obtained native May1 crystal seeds. After 9 days at 25 °C, cocrystals used for X-ray data collection were harvested, flash-frozen in liquid nitrogen with the reservoir solution as self-cryoprotectant and stored. These conditions yielded crystals of space group *C*222_1_ (*a* = 97.17 Å, *b* = 113.39 Å, *c* = 91.22 Å, α = β = γ = 90°) diffracting up to 1.81 Å.

Diffraction data for the May1–**25** complex was collected at 100 K on an in-house MicroMax-007 HF Microfocus X-ray generator with a VariMax VHF ArcSec confocal optical system (Rigaku, Japan), an AFC11 partial four-axis goniometer (Rigaku, Japan), a PILATUS 300 K detector (Dectris, Switzerland), and a Cryostream 800 cryocooling system (Oxford Cryosystems, England). Diffraction data were processed using XDS.^100^ The crystal parameters and data collection and refinement statistics are summarized in Table S1.

### Structure Determination, Model Building, and Refinement

The structure of the May1–**25** complex was solved by molecular replacement with CCP4 Molrep^101^ using the native May1 structure (PDB 6R5H(17)) as a search model. The initial models were refined through several cycles of manual building using Coot and automated refinement with CCP4 REFMAC5.^102^ Visualization of structural data was performed in PyMOL 0.99rc6, and 2D diagrams summarizing molecular interaction between inhibitors and May1 were prepared using LigPlot.^103^ Atomic coordinates and structure factors were deposited into the Protein Data Bank under code 6R61.

### Molecular Modeling

The crystal structure of May1–**25** complex was preprocessed using the Protoss tool within the ProteinsPlus web service, which automatically predicts tautomers and protonation states in protein–ligand complexes.^30^ A second protonation variant of the complex was prepared using the Protein Preparation Workflow in Maestro (ver. 2024-1) with the OPLS4 force field and PROPKA at a pH of 7.4.^31^ Compound **21** was modeled from the preprocessed complex of May1–**25** complex by modifying 3-pyridylalanine to valine.

The protein structure was converted into a PDB format compatible with AMBER by selecting a single conformer (A) for all atoms with alternate conformations, renumbering residues starting at 1, identifying disulfide bonds, and correctly naming protonated histidines (CYX, HIE, HID). Missing hydrogens were added, and termini were capped appropriately. The structurally significant water molecule Wat175 was retained as part of the protein structures.

All hydrogens were relaxed through optimization and simulated annealing (300 to 0 K over 60 ps), and the protein–ligand complexes were gradually relaxed using a series of optimizations following our previous protocol.^20^ The SQM2.20 scoring function was used to calculate stabilization energies and final scores, incorporating gas-phase interaction energy (Δ*E*_int_), the change of solvation free energy upon complex formation (ΔΔ*G*_solv_), the change in ligand conformational free energy in an aqueous environment [Δ*G*_conf_(L)] and the loss of ligand conformational entropy (*T*Δ*S*) upon binding.^20^

### Binding “Free” Energy Contributions

Binding contributions of the inhibitor’s structural components were elucidated using ligand fragmentation coupled with SQM-based scoring^20^ at the PM6-D3H4/COSMO2 level.^32−34^ The inhibitor was fragmented into seven distinct parts by cleaving C–C bonds and capping the resulting termini with hydrogens. The binding “free” energy contribution of each fragment (comprising gas-phase energy and solvation free energy components) was calculated as the energy difference between the entire bound compound **25** and the complex missing the specific fragment.

### Cytotoxicity Assay

Acute lymphoblastic leukemia (CCRF-CEM), acute promyelocytic leukemia (HL-60), cervix cancer (HeLa), and breast adenocarcinoma (MCF-7) human cell lines were purchased from ATCC (LGC Standards). HeLa cells were cultured in DMEM High Glucose medium, CCRF-CEM and HL-60 cells in RPMI-1640 medium (Dutch modification), and MCF 7 cells in MEM medium. All media were supplemented with 10% (v/v) heat inactivated fetal bovine serum, and 2 mM glutamine at 37 °C in a humidified atmosphere containing 5% CO_2_. Twice a week, when the adherent cells reached up to 80–90% of confluency, they were subcultured using 0.25% trypsin/1 mM EDTA solution for further passage. The concentrations of CCRF-CEM and HL-60 cell suspensions were maintained below 1,500,000 cells/mL and 2,000,000 cells/mL, respectively.

CellTiter-Glo Luminescent Cell Viability Assay (Promega) was used to determine the cytotoxicity of compounds **9** and **25**, as well as selected reference antifungal drugs. After seeding into white 384-well plates (Thermo Nunc 164610), the cells (20 μL) were grown for 24 h before adding compounds or DMSO (vehicle control) into each well. After 72 h treatment, CellTiter-Glo reagent (20 μL) was added to each well, and the plate was mixed for 2 min at 400 rpm on an orbital shaker in the dark. The luminescent signal was then allowed to stabilize for 10 min at room temperature. Luminescence, which directly correlates with the cell number, was recorded using a microplate luminometer (BioTek Cytation 3). The CC_50_ values were calculated by nonlinear regression analysis of the normalized data, assuming a sigmoidal concentration response curve with variable Hill slope (GraphPad Software GraphPad Prism 7).

### Plasma Stability Assay

The plasma stability of the compounds was evaluated by incubating 5 μM solutions with human pooled plasma from 50 donors (Biowest) at 37 °C for 20, 60, and 120 min. To terminate the reactions, four volumes of ice-cold methanol were added. The samples were then thoroughly mixed, stored at −20 °C for 30 min and left overnight at 8 °C. Before analysis, the samples were centrifuged at 2000*g* at 8 °C for 20 min. The supernatants were diluted with four volumes of 30% methanol in water and analyzed using the Echo MS system (SCIEX). Zero time points were prepared by mixing the compounds with methanol prior to adding the plasma.

### Microsomal Stability Assay

The stability of the compounds in human liver microsomes was evaluated using 0.5 mg/mL pooled microsomes (Thermo Fisher Scientific) and 5 μM compounds in 90 mM Tris-HCl buffer (pH 7.4) supplemented with 2 mM NADPH and 2 mM MgCl_2_. Incubations were conducted at 37 °C for 10, 30, and 45 min. Reactions were stopped by adding four volumes of ice-cold methanol, followed by vigorous mixing and storage at −20 °C for 30 min and left overnight at 8 °C. After centrifugation, the supernatants were diluted with four volumes of 30% methanol in water and analyzed using the Echo MS system (SCIEX). Zero time points were created by premixing the compounds and cofactors with methanol before adding the microsomes.

### Caco-2 Permeability Assay

The bidirectional transepithelial transport of 5 μM test compounds at pH 7.4 across Caco-2 cell monolayers was assessed using the BD BioCoat HTS Caco-2 assay system (BD Biosciences, Bedford, MA) according to the manufacturer’s protocol. At the conclusion of the 3-h transport period, aliquots were taken from both the donor and acceptor compartments. The integrity of the Caco-2 monolayers was confirmed using Lucifer Yellow dye, and compound concentrations were determined with LC–MS/MS (Sciex 6500 triple quadrupole). The apparent permeability coefficient (*P*_app_) was calculated as follows: , where  represents the rate of compound absorption, *C*_0_ is the initial donor concentration, and *A* is the monolayer surface area. The efflux ratio was calculated as the ratio of permeability in the basolateral-to-apical direction (*P*_app_ B → A) to that in the apical-to-basolateral direction (*P*_app_ A → B).

### Pharmacokinetics

Male C57BL6/N mice (Charles River Laboratories) were housed under controlled conditions with a 12-h light/dark cycle, ambient temperature maintained at 22 ± 3 °C, and relative humidity of 50 ± 20%. Animals were fasted for 4 h prior to dosing but had unrestricted access to water throughout the study. All animal studies were ethically reviewed and performed in accordance with European directive 2010/63/EU and were approved by the Czech Central Commission for Animal Welfare project of experiments 34/2019/CZ. A dose of 10 mg/kg was administered intravenously via tail vein or via oral gavage in 5% DMSO, 30% PEG and saline for **9**, or 30% PEG and saline for **25**. Blood samples were collected under anesthesia via the retro-orbital venous sinus at time points of 5, 15, and 30 min and 1, 2, and 4 h postdose. Samples were processed and stored at −70 °C until further analysis. To precipitate proteins, one volume of plasma was mixed with three volumes of ice–cold methanol, followed by vigorous mixing and stored at −20 °C overnight. Before analysis, the samples were centrifuged at 20,000*g* at 8 °C for 10 min.

The supernatants were analyzed using LC–MS/MS. Calibration standards were prepared from a 1 mg/mL stock solution in 80% ACN. LC separation was performed on a Synergi 4 μm Fusion 50 × 2 mm column (Phenomenex) with a water/ACN gradient containing 0.1% formic acid, increasing from 5% to 90% over 8 min. MS/MS analysis was conducted on a QTRAP 6500 (Sciex 6500 triple quadrupole) using multiple reaction monitoring. Plasma concentrations were measured to generate concentration vs time profiles, and the area under the curve (AUC) was calculated using the linear trapezoidal method. Statistical analyses were performed using PK Solver 2.0.^35^

### Indole Resin Loading

Indole resin (150 mg, 0.122 mmol, Iris BR-5218) and 3 Å molecular sieves (5 g, Merck 208574) were suspended in anhydrous MeOH (5 mL), and a solution of aliphatic amino acid (2.43 mmol) fully dissolved in glacial acetic acid (7.5 mL) was added. All amino acids were obtained from commercial sources except for 14-aminotetradecanoic acid, preparation of which we described previously,^11^ 13-aminotridecanoic acid (**S1**), which was prepared by Gabriel synthesis,^36^ and 15-aminopentadecanoic acid (**S2**), which was prepared by Beckmann rearrangement with subsequent hydrolysis.^37^ Details of the amino acid syntheses are included in the Supporting Information. The reaction mixture was incubated at 37 °C in a 50 mL PP centrifuge tube for 18 h. This step requires mixing the suspension which was done by shaking in horizontal position. The resin suspension was then separated from the molecular sieves by decanting and repeated washing of the remaining sieves with the collected supernatant. A solution of NaBH_3_CN (229 mg, 3.65 mmol) in anhydrous methanol (2.5 mL) was then added to the suspension, resulting in hydrogen evolution. The reaction mixture was stirred at room temperature for 18 h in 50 mL PP centrifuge tube pierced with a 25 g needle.

The resin was then collected by centrifugation, transferred to a PP frit column, washed with methanol until the sieve dust was removed and dried in vacuo to a constant weight. Dried resin was washed with DMF to achieve full swelling and a solution of Fmoc-Succinimide (123 mg, 0.365 mmol) and DIEA (191 μL, 1.10 mmol) in DMF (4 mL) was added. The reaction mixture was incubated with mixing by shaking at room temperature for 2 h. The resin was washed with 2 × 10 mL DMF, 2 × 10 mL MeOH and 4 × 10 mL DCM. It was then treated with a solution of TrtCl (68 mg, 0.243 mmol) and DIEA (212 μL, 1.22 mmol) in DCM (5 mL) for 18 h at room temperature with shaking. Finally, the protected resin was washed with 4 × 10 mL DCM, and 4 × 10 mL MeOH, then dried in vacuo to a constant weight.

### Macrocyclic Library Assembly

The synthesis of macrocyclic inhibitors was conducted on 384-well filter plates (Cytiva 5072-N) with a reaction scale of 250 nmol. The amount of resin per well was adjusted based on the determined resin loading (Figure S1). Incubations were carried out using an apparatus that alternated between N_2_ overpressure (0.05 atm) and suction (−0.75 atm) to mix the well contents, with a period of 5 s between 200 ms suction pulses.

Deprotection was performed by sequential addition of 6 × 40 μL of 20% piperidine in DMF, with each addition incubated for 5 min. Wells were washed using 35, 80, and 6 × 35 μL of DMF. Supernatants were removed via suction applied for 1 min.

First, the P1′ residue was coupled using 2 × 45 μL of 150 mM protected amino acid, 188 mM Oxyma Pure, and 188 mM DIC in DMF incubated for 3 h, followed by washing, deprotection and washing. The P1 phenylstatine residue was coupled using 2 × 45 μL of 50 mM protected amino acid, 63 mM Oxyma Pure, and 63 mM DIC in DMF for 2 h, followed by washing and deprotection. Wells were then washed and incubated with 2 × 45 μL of AcOH/MeOH/TIS (80:15:5) for 30 min, washed with 70 μL DCM, and subjected to suction for 10 min to remove residual solvent.

The apparatus was disassembled and thoroughly washed with DMF. The filter plate was placed on a receiving 384-well plate and centrifuged for 10 min at 2000*g* to dry it. The apparatus was reassembled, and the plate was washed with 4 × 35 μL DMF, 2 × 70 μL DCM, and again with 4 × 35 μL DMF. The plate was then left under suction for 10 min.

Macrocyclization was effected by incubating the resin with 80 μL of 13.3 mM HCTU (5 equiv) and 1.3% DIEA (15 equiv) in DMF for 24 h. Following the reaction, the plate was washed sequentially with 4 × 35 μL DMF, 4 × 70 μL DCM, and 4 × 70 μL MeOH, and the apparatus was disassembled. The filter plate was placed onto a receiving 384-well plate and centrifuged at 2000*g* for 10 min to dry the plate.

To cleave and deprotect the macrocycles, the filter plate was transferred to a clean deep-well receiving plate (Merck BR701355). A mixture of 95% TFA and 5% water was added sequentially in 30 μL aliquots at 0, 15, 45, 100, and 120 min. The plate was then centrifuged at 2000*g* for 10 min to collect the cleavage mixture. The collected solution was evaporated overnight under a stream of N_2_ from a 384-needle manifold. Finally, the dried residues were dissolved in 25 μL of DMSO, yielding a 10 mM stock solution of the macrocyclic inhibitors.

Purity testing for library compounds was conducted on an Agilent 1290 Infinity II LC/MSD System with a ZORBAX RRHT StableBond C18 (80 Å, 2.1 × 50 mm, 1.8 μm) column using 0.5–98% ACN gradient elution and 0.1% formic acid as additive. Evaporative light scattering detection was used for quantitation to account for differences in UV absorption among library members. Raw data were evaluated automatically using Mnova QtScript.^38^

### General Procedures

Commercially available HPLC-grade ACN, catalysts, and reagent-grade chemicals were used without further purification. Thin-layer chromatography (TLC) was conducted on silica gel 60 F254-coated aluminum sheets (Merck). Flash chromatography was performed with silica gel 60 (particle size 0.040–0.063 mm, Fluka). Chemicals were obtained from Sigma-Aldrich, TCI, Enamine, BroadPharm, BLD Pharm, and Iris Biotech.

NMR spectra were recorded at room temperature, unless otherwise specified, using a 400 MHz Bruker AVANCE III HD. Low-resolution ESI mass spectra were recorded using a quadrupole orthogonal acceleration time-of-flight (Q-ToF micro, Waters) mass spectrometer, while high-resolution ESI mass spectra were obtained on an LTQ Orbitrap XL hybrid FT mass spectrometer (Thermo Fisher Scientific). Ionization conditions in the ESI Orbitrap source were optimized as follows: sheath gas flow rate of 35 au, auxiliary gas flow rate of 10 au (nitrogen), source voltage of 4.3 kV, capillary voltage of 40 V, capillary temperature of 275 °C, and tube lens voltage of 155 V. Samples were dissolved in methanol and introduced via direct injection.

Inhibitors were purified using preparative-scale HPLC on a JASCO PU-975 system (10 mL min^–1^ flow rate), equipped with a UV-975 detector and a Waters YMC-PACK ODS-AM C18 preparative column (5 μm, 20 × 250 mm). Analytical HPLC was performed on a JASCO PU1580 system (1 mL min^–1^ flow rate, gradient elution from 2 to 100% ACN over 30 min) using a Watrex C18 analytical column (5 μm, 250 × 5 mm) to verify compound purity. All final inhibitors achieved a minimum purity of 95% (LC, Figure S5). Compounds **1**–**8**, and **9**–**24** were prepared as described previously.^11^ All assayed compounds passed the PAINS filter using false positive remover.^39^

#### Methyl (*S*)-12-(2-(((Benzyloxy)carbonyl)amino)-6-((tert-butoxycarbonyl)amino)hexanamido) Dodecanoate (**9a**)

Cbz-Lys(Boc)-OH (157 mg, 0.41 mmol) and HATU (150 mg, 0.40 mmol) were dissolved in anhydrous DMF (3 mL), the reaction mixture was cooled down to 0 °C and DIEA (200 μL, 1.13 mmol) was added. The reaction mixture was stirred at 0 °C for 5 min and then solution of methyl 12-aminododecanoate hydrochloride (100 mg, 0.38 mmol) in anhydrous DMF (2 mL) was added. The reaction mixture was stirred at room temperature for 6 h under inert atmosphere. The reaction mixture was diluted with CHCl_3_ (30 mL) and washed with saturated NaHCO_3_ (2 × 25 mL), 10% KHSO_4_ (2 × 25 mL), brine (35 mL), dried over MgSO_4_ and the organic solvent was evaporated in vacuo. The column chromatography (cyclohexane–EtOAc 1:1) afforded desired product **9a** (180 mg, 81%) as a colorless amorphous solid.

^**1**^**H NMR** (401 MHz, CD_3_OD): 1.23–1.38 (m, 16H), 1.44 (s, 9H), 1.46–1.55 (m, 4H), 1.55–1.81 (m, 4H), 2.32 (t, *J* = 7.4 Hz, 2H), 2.99–3.09 (m, 2H), 3.11–3.25 (m, 2H), 3.66 (s, 3H), 4.05 (dd, *J* = 8.9, 5.4 Hz, 1H), 5.10 (s, 2H), 6.56 (bs, 1H), 7.29–7.41 (m, 5H).

^**13**^**C NMR** (101 MHz, CD_3_OD): 22.78, 24.64, 26.52, 27.42, 28.79, 28.96, 28.97, 29.00, 29.17, 29.22, 29.26, 31.69, 33.41, 38.98, 39.60, 50.57, 55.22, 66.28, 78.46, 127.48, 127.64, 128.09, 136.78, 157.00, 157.18, 173.42, 174.61.

**ESI MS**: 614 ([M + Na]^+^).

**HR ESI MS**: calcd for C_32_H_53_N_3_O_7_Na^+^, 614.37757; found, 614.37737.

#### Methyl (*S*)-12-(2-(((Benzyloxy)carbonyl)amino)-6-((methoxycarbonyl)amino)hexanamido)dodecanoate (**9b**)

Compound **9a** (150 mg, 0.25 mmol) was dissolved in DCM (2 mL) and TFA (2 mL) was added. The reaction mixture was stirred at room temperature for 1 h. Next, volatiles were removed in vacuo and the residue was dissolved in anhydrous DCM (5 mL) under inert atmosphere. Then, DIEA (177 μL, 1.01 mmol) and methyl chloroformate (29 μL, 0.38 mmol) were added. The reaction mixture was stirred at room temperature for 30 min. Next, the reaction mixture was diluted with CHCl_3_ (30 mL) and washed with saturated NaHCO_3_ (2 × 25 mL), 10% KHSO_4_ (2 × 25 mL), and brine (35 mL). The organic solvent was dried over MgSO_4_ and evaporated in vacuo to afford the desired product **9b** (140 mg, quant.) as a colorless amorphous solid.

^**1**^**H NMR** (401 MHz, CD_3_OD): δ 1.22–1.55 (m, 20H), 1.55–1.83 (m, 4H), 2.32 (t, *J* = 7.4 Hz, 2H), 3.05–3.13 (m, 2H), 3.09–3.24 (m, 2H), 3.61 (s, 3H), 3.66 (s, 3H), 4.06 (dd, *J* = 8.9, 5.4 Hz, 1H), 5.10 (s, 2H), 6.82 (t, *J* = 5.9 Hz, 1H), 7.26–7.41 (m, 5H), 7.96 (t, *J* = 5.7 Hz, 1H).

^**13**^**C NMR** (101 MHz, CD_3_OD): 22.70, 24.67, 26.57, 28.83, 29.00, 29.01, 29.04, 29.13, 29.21, 29.27, 29.30, 31.71, 33.47, 39.04, 40.04, 50.66, 51.07, 55.19, 66.33, 127.51, 127.68, 128.13, 136.76, 156.96, 158.21, 173.34, 174.60.

**ESI MS**: 572 ([M + Na]^+^).

**HR ESI MS**: calcd for C_29_H_47_N_3_O_7_Na^+^, 572.33062; found, 527.33036.

#### Methyl (6*S*,7*S*,11*S*)-6-Benzyl-7-hydroxy-11-(4-((methoxycarbonyl)amino)butyl)-2,2-dimethyl-4,9,12-trioxo-3-oxa-5,10,13-triazapentacosan-25-oate (**9c**)

Compound **9b** (140 mg, 0.26 mmol) was dissolved in THF (5 mL) and 10% Pd/C (14 mg) was added. The reaction mixture was stirred under H_2_ atmosphere for 90 min. Then, Pd/C was removed by filtration and the solvent was evaporated in vacuo. Next, Boc-phenylstatine (91 mg, 0.29 mmol) and HBTU (90 mg, 0.28 mmol) were dissolved in anhydrous DMF (2 mL) and DIEA (177 μL, 1.02 mmol) was added. The reaction mixture was stirred at room temperature for 5 min and a solution of the residue from the hydrogenation in anhydrous DMF (1.5 mL) was added. The reaction mixture was stirred at room temperature for 12 h under inert atmosphere. The organic solvent was then evaporated in vacuo. The residue was chromatographed on silica gel (gradient CHCl_3_–MeOH 30:1 → 20:1) to obtain the desired product (150 mg, 83%) as an amorphous solid.

^**1**^**H NMR** (401 MHz, CD_3_OD): 1.27–1.34 (m, 16H), 1.36 (s, 9H), 1.44–1.56 (m, 4H), 1.56–1.72 (m, 3H), 1.75–1.86 (m, 1H), 2.32 (t, *J* = 7.4 Hz, 2H), 2.42–2.55 (m, 2H), 2.75–2.94 (m, 2H), 3.06–3.13 (m, 2H), 3.13–3.21 (m, 2H), 3.64 (s, 3H), 3.66 (s, 3H), 3.71–3.83 (m, 1H), 4.11 (td, *J* = 7.1, 1.9 Hz, 1H), 4.17–4.27 (m, 1H), 6.25 (d, *J* = 9.7 Hz, 1H), 6.86 (t, *J* = 5.9 Hz, 1H), 7.13–7.31 (m, 5H), 8.05 (d, *J* = 7.0 Hz, 1H), 8.11 (t, *J* = 5.9 Hz, 1H).

^**13**^**C NMR** (101 MHz, CD_3_OD): 22.61, 24.65, 26.60, 27.42, 28.81, 28.97, 29.00, 29.03, 29.16, 29.18, 29.26, 29.28, 31.08, 33.43, 38.03, 39.04, 39.96, 40.07, 50.59, 51.06, 53.88, 55.33, 69.38, 78.71, 125.78, 127.86, 128.98, 138.84, 156.95, 158.27, 172.54, 172.95, 174.59.

**ESI MS**: 729 ([M + Na]^+^).

**HR ESI MS**: calcd for C_37_H_62_N_4_O_9_Na^+^, 729.44090; found, 729.44036.

#### Methyl (4-((3*S*,7*S*,8*S*)-8-Benzyl-7-hydroxy-2,5,10-trioxo-1,4,9-triazacyclohenicosan-3-yl) butyl)carbamate (**9**)

Methyl ester **9c** (50 mg, 0.07 mmol) was dissolved in THF (4 mL) and H_2_O (3 mL) and LiOH (17 mg, 0.71 mmol) was added. The reaction mixture was stirred at room temperature for 2 h, then acidified with 1 M hydrochloric acid (5 mL). CHCl_3_ (10 mL) was added, the layers were separated and the product was extracted with additional CHCl_3_ (3 × 10 mL). Organic layers were combined, dried over MgSO_4_ and the solvent was removed in vacuo to yield the crude acid. Next, the residue was dissolved in DCM (2 mL) and TFA (2 mL) was added. The reaction mixture was stirred at room temperature for 1 h. Next, volatiles were removed in vacuo. The residue was then dissolved in anhydrous DMF (2 mL) and DIEA (55 μL, 0.32 mmol) was added. This solution was added over 8 h to a solution of PyBOP (43 mg, 0.08 mmol) in anhydrous DMF (5 mL). The reaction mixture was stirred overnight and evaporated. Crude product **9** was purified by preparative HPLC (19 mg, 46% over 3 steps).

^**1**^**H NMR** (401 MHz, DMSO-*d*_6_): δ 0.93–1.05 (m, 2H), 1.06–1.52 (m, 21H), 1.58–1.71 (m, 1H), 2.03–2.17 (m, 2H), 2.17–2.31 (m, 2H), 2.66–2.84 (m, 2H), 2.94 (q, *J* = 6.6 Hz, 2H), 3.18–3.41 (m, 2H), 3.51 (s, 3H), 3.84–3.94 (m, 1H), 3.94–4.05 (m, 2H), 5.03 (bs, 1H), 7.08 (t, *J* = 5.7 Hz, 1H), 7.11–7.27 (m, 5H), 7.66 (d, *J* = 9.2 Hz, 1H), 7.82 (d, *J* = 7.4 Hz, 1H), 7.99 (t, *J* = 4.6 Hz, 1H).

^**13**^**C NMR** (101 MHz, DMSO-*d*_6_): δ 23.39, 25.27, 25.32, 26.47, 27.02, 27.15, 27.49, 27.56, 27.76, 28.16, 29.63, 31.50, 35.26, 37.39, 38.74, 40.25, 40.55, 51.59, 53.78, 53.92, 69.94, 126.24, 128.39, 129.41, 139.99, 157.13, 170.87, 172.04, 172.92.

**ESI MS**: 597 ([M + Na]^+^).

**HR ESI MS**: calcd for C_31_H_50_N_4_O_6_Na^+^, 597.36226; found, 597.36198.

#### Methyl (*S*)-14-(2-((*t*-Butoxycarbonyl)amino)-3-(pyridin-3-yl)propanamido) Tetradecanoate (**25a**)

Boc-(*L*)-3Pal-OH (136 mg, 0.51 mmol) and HBTU (194 mg, 0.51 mmol) were dissolved in anhydrous DMF (3 mL) and DIEA (260 μL, 1.49 mmol) was added. The reaction mixture was stirred at room temperature for 30 min and a solution of methyl 14-aminotetradecanoate (110 mg, 0.42 mmol, prepared as previously described^11^) in anhydrous DMF (2 mL) was added. The reaction mixture was stirred at room temperature for 6 h under inert atmosphere. The reaction mixture was diluted with CHCl_3_ (30 mL) and washed with saturated NaHCO_3_ (2 × 25 mL), 10% KHSO_4_ (2 × 25 mL), and brine (35 mL). The mixture was dried over MgSO_4_, and the organic solvent was evaporated in vacuo. Flash chromatography (CHCl_3_–MeOH 35:1) afforded desired product **25a** (110 mg, 51%) as an amorphous solid.

^**1**^**H NMR** (400 MHz, CDCl_3_): 1.10–1.31 (27H, m), 1.41–1.59 (4H, m), 2.23 (2H, t, *J* = 7.5), 2.97–3.08 (1H, m), 3.11–3.22 (2H, m), 3.36–3.48 (1H, m), 3.59 (3H, s), 4.61–4.71 (1H, m), 5.92 (1H, d, *J* = 6.7), 7.63–7.76 (2H, m), 8.19–8.29 (1H, m), 8.53–8.63 (1H, m), 9.16 (1H, s).

^**13**^**C NMR** (101 MHz, CDCl_3_): 24.93, 26.99, 28.17 (3C), 29.12, 29.23, 29.29, 29.35, 29.42, 29.55, 29.56, 29.58, 29.59, 34.09, 37.01, 39.79, 51.42, 54.73, 79.83, 125.89, 138.78, 139.46, 143.24, 145.96, 155.35, 170.26, 174.32.

**ESI MS**: 528 ([M + Na]^+^).

**HR ESI MS**: calcd for C_28_H_47_O_5_N_3_Na, 528.34079; found, 528.34063.

#### Methyl (*S*)-14-(2-Amino-3-(pyridin-3-yl)propanamido)tetradecanoate (**25b**)

Boc-aminoester **25a** (100 mg, 0.19 mmol) was dissolved in dichloromethane (5 mL) and TFA (0.5 mL) was added. The reaction mixture was stirred at room temperature for 1 h. The mixture was diluted with DCM (10 mL), washed with saturated NaHCO_3_ (2 × 10 mL), and dried over MgSO_4_. The organic solvent was evaporated in vacuo to obtain crude amine **25b** (70 mg, 87%).

**ESI MS**: 406 ([M + H]^+^).

#### Methyl (6*S*,*7S*,*11S*)-6-Benzyl-7-hydroxy-2,2-dimethyl-4,9,12-trioxo-11-(pyridin-3-ylmethyl)-3-oxa-5,10,13-triazaheptacosan-27-oate (**25c**)

Boc-phenylstatine (93 mg, 0.30 mmol) and HBTU (114 mg, 0.30 mmol) were dissolved in anhydrous DMF (2 mL) and freshly distilled DIEA (174 μL, 1.00 mmol) was added. The reaction mixture was stirred at room temperature for 30 min and a solution of amine **25b** (130 mg, 0.25 mmol) in anhydrous DMF (1.5 mL) was added. The reaction mixture was stirred at room temperature for 12 h under inert atmosphere. The organic solvent was evaporated in vacuo. The residue was chromatographed on silica gel (CHCl_3_–MeOH 15:1) to obtain the desired product **25c** (95 mg, 54%) as an amorphous solid.

^**1**^**H NMR** (400 MHz, CD_3_OD): 1.20–1.33 (18H, m), 1.34 (9H, s), 1.37–1.45 (2H, m), 1.55–1.64 (2H, m), 2.31 (2H, t, *J* = 7.4), 2.72–2.97 (3H, m), 3.05–3.22 (3H, m), 3.30–3.33 (2H, m), 3.65 (3H, s), 3.72–3.79 (1H, m), 3.98–4.05 (1H, m), 4.60 (1H, dd, *J* = 8.7, *J* = 5.8), 7.19–7.27 (5H, m), 7.35 (1H, dd, *J* = 7.8, *J* = 4.9), 7.75 (1H, dt, *J* = 7.8, *J* = 1.8), 8.39 (1H, dd, *J* = 4.9, *J* = 1.5), 8.43 (1H. d, *J* = 1.7).

^**13**^**C NMR** (101 MHz, CD_3_OD): 26.01, 27.93, 28.77 (3C), 30.17, 30.28, 30.36, 30.39, 30.57, 30.65, 30.68, 30.71 (2C), 34.79, 35.95, 39.13, 40.51, 41.38, 51.95, 55.67, 56.82, 70.53, 80.05, 125.08, 127.14, 129.22 (2C), 130.33 (2C), 135.21, 138.99, 140.13, 148.35, 150.76, 158.30, 172.68, 173.69, 175.93.

**ESI MS**: 719 ([M + Na]^+^).

**HR ESI MS**: calcd for C_39_H_60_O_7_N_4_Na, 719.43542; found, 719.43549.

#### (6*S*,7*S*,11*S*)-6-Benzyl-7-hydroxy-2,2-dimethyl-4,9,12-trioxo-11-(pyridin-3-ylmethyl)-3-oxa-5,10,13-triazaheptacosan-27-oic Acid (**25d**)

To a solution of methylester **25c** (87 mg, 0.12 mmol) in THF (2 mL), a solution of 1 M LiOH (2 mL) was added dropwise. The reaction mixture was stirred at room temperature for 2 h, then acidified with 1 M hydrochloric acid (5 mL) and CHCl_3_ (10 mL) was added. Layers were separated and the product was extracted with CHCl_3_ (3 × 10 mL). Organic layers were combined, dried over MgSO_4_, and the solvent was removed in vacuo. The residue was chromatographed on silica gel (CHCl_3_–MeOH) to afford desired acid **25d** (60 mg, 70%).

^**1**^**H NMR** (400 MHz, CD_3_OD): 1.21–1.32 (18H, m), 1.33 (9H, s), 1.36–1.45 (2H, m), 1.54–1.63 (2H, m), 2.27 (2H, t, *J* = 7.4), 2.31–2.41 (2H, m), 2.75 (1H, dd, *J* = 13.7, *J* = 9.4), 2.82–2.96 (2H, m), 3.03–3.21 (3H, m), 3.69–3.79 (1H, m), 3.97–4.02 (1H, m), 4.58 (1H, dd, *J* = 8.7, *J* = 5.8), 6.25 (1H, d, *J* = 9.7), 7.13–7.18 (1H, m), 7.20–7.27 (5H, m), 7.36 (1H, dd, *J* = 7.8, *J* = 4.9), 7.75 (1H, dt, *J* = 7.8, *J* = 1.8), 8.07 (1H, t, *J* = 5.6), 8.39 (1H, dd, *J* = 4.9, *J* = 1.3), 8.42 (1H, d, *J* = 1.7).

^**13**^**C NMR** (101 MHz, CD_3_OD): 26.11, 27.94, 28.77 (3C), 30.24, 30.30, 30.41, 30.42, 30.61, 30.66, 30.71, 30.72, 30.73, 34.98, 35.96, 39.14, 40.53, 41.39, 55.70, 56.85, 70.55, 80.09, 125.13, 127.16, 129.24 (2C), 130.34 (2C), 135.28, 139.08, 140.15, 148.32, 150.72, 158.34, 172.72, 173.73, 177.71.

**ESI MS**: 705 ([M + Na]^+^).

**HR ESI MS**: calcd for C_38_H_58_O_7_N_4_Na, 705.41977; found, 705.41989.

#### (3*S*,7*S*,8*S*)-8-Benzyl-7-hydroxy-3-(pyridin-3-ylmethyl)-1,4,9-triazacyclotricosane-2,5,10-trione (**25**)

To a solution of acid **25d** (60 mg, 0.08 mmol) and TSTU (0.08 mmol) in anhydrous DMF (2 mL), DIEA (0.08 mmol) was added. The reaction mixture was stirred at room temperature for 2 h under inert atmosphere. The reaction mixture was diluted with CHCl_3_ (15 mL), washed with brine (2 × 10 mL), and dried over MgSO_4_. The organic solvent was evaporated in vacuo. The residue was dissolved in TFA (1 mL) and the reaction mixture was stirred at room temperature for 15 min. TFA was removed by flow of nitrogen to obtain crude amine. Crude amine was dissolved in anhydrous DMF (5 mL) and added to a solution of DIEA (5 mL) in anhydrous DMF (20 mL). The reaction mixture was stirred overnight and evaporated. Crude product **25** was purified by preparative HPLC (17 mg, 41% over 3 steps).

^**1**^**H NMR** (400 MHz, CD_3_OD): 1.23–1.33 (18H, m), 1.42–1.51 (4H, m), 2.15 (2H, td, *J* = 6.9, *J* = 2.4), 2.32 (2H, dd, *J* = 7.1, *J* = 2.5), 2.80 (1H, dd, *J* = 13.8, *J* = 10.1), 2.89 (1H, dd, *J* = 13.8, *J* = 4.9), 2.97 (1H, dt, *J* = 13.4, *J* = 6.9), 3.10 (1H, dd, *J* = 14.3, *J* = 9.4), 3.33–3.39 (1H, m), 3.45 (1H, dd, *J* = 14.3, *J* = 5.3), 4.01–4.08 (2H, m), 4.71 (1H, dd, *J* = 9.3, *J* = 5.3), 7.14–7.26 (5H, m), 8.00 (1H, dd, *J* = 7.9, *J* = 5.9), 8.53 (1H, d, *J* = 8.1), 8.74 (1H, d, *J* = 5.4), 8.77 (1H, s).

^**13**^**C NMR** (101 MHz, CD_3_OD): 26.68, 27.10, 28.26, 28.50, 28.66, 28.83, 28.87, 29.24, 29.28, 29.57, 29.87, 35.62, 37.07, 38.57, 40.76, 41.43, 54.98, 55.43, 70.96, 127.34, 128.13, 129.31 (2C), 130.22 (2C), 140.18 (2C), 141.15, 143.28, 148.60, 172.16, 173.56, 176.06.

**ESI MS**: 587 ([M + Na]^+^).

**HR ESI MS**: calcd for C_33_H_48_O_4_N_4_Na, 587.35678; found, 587.35663.

## Supporting Information Available

Procedures for aliphatic amino acids synthesis, resin loading efficiency, analysis of library purity, raw inhibition curves for May1 and off-target enzymes, analytical HPLC traces for key compounds, data collection and refinement statistics, in silico model protonation state comparison, in silico comparison of binding affinity for **21** and **25**, interaction “free” energy for **25** with individual fragments removed and with individual fragments in isolation, plasma and microsomal stability, Caco-2 permeability assay evaluation, and pharmacokinetic properties of **9** and **25**. The following files are available free of charge. SI macrocyclic inhibitors May1 (PDF)
SMILES list macrocyclic inhibitors May1 (CSV)

## Abbreviations

3Pal: 3-(pyridin-3-yl)-l-alanine
4Pal: 3-(pyridin-4-yl)-l-alanine
Aad: l-2-aminoadipic acid
Abu: l-2-aminobutyric acid
ACN: acetonitrile
AlaPyz: 3-(pyrazol-1-yl)-l-alanine
Cit: l-citrulline
C*x*: unbranched aliphatic *x*-amino acid (e.g., C6: 6-aminohexanoic acid)
CysEt: *S*-ethyl-l-cysteine
Dab: l-2,4-diaminobutanoic acid
Dap: l-2,3-diaminopropionic acid
DCM: dichloromethane
DIC: *N*,*N*′-diisopropylcarbodiimide
DIEA: *N*,*N*-diisopropylethylamine
DMF: *N*,*N*-dimethylformamide
DmGlu: 3,3-dimethyl-l-glutamic acid
DMSO: dimethyl sulfoxide
DOOG: 14-amino-5-oxo-3,9,12-trioxa-6-azatetradecanoic acid
Eth: l-ethionine
HBTU: 2-(1*H*-benzotriazol-1-yl)-1,1,3,3-tetramethyluronium hexafluorophosphate
HCTU: 2-(6-chloro-1*H*-benzotriazole-1-yl)-1,1,3,3-tetramethyluronium hexafluorophosphate
Hph: l-homophenylalanine
Cha: 3-cyclohexyl-l-alanine
LysAc: *N*-ε-acetyl-l-lysine
LysDNP: *N*-ε-(2,4-dinitrophenyl)-l-lysine
May1: major aspartyl peptidase 1
MES: 2-(*N*-morpholino)ethanesulfonic acid
Nle: l-norleucine
Nva: l-norvaline
Omh: *O*-methyl-l-homoserine
Oms: *O*-methyl-l-serine
Omt: *O*-methyl-l-threonine
Orn: l-ornithine
Pst: (3*S*,4*S*)-4-amino-3-hydroxy-5-phenylpentanoic acid
Sem: l-selenomethionine
TFA: trifluoroacetic acid
THF: tetrahydrofuran
TIS: triisopropylsilane
TMOF: trimethyl orthoformate
TrtCl: triphenylmethyl chloride (trityl chloride)
TSTU: *N*,*N*,*N*′,*N*′-tetramethyl-*O*-(*N*-succinimidyl)uronium tetrafluoroborate
TTDS: 1-amino-15-oxo-4,7,10-trioxa-14-azaoctadecan-18-oic acid
TyrNO_2_: 3-nitro-l-tyrosine
YNB: yeast nitrogen base
