# Supplementary material for: On-Resin Assembly of Macrocyclic Inhibitors of Cryptococcus neoformans May1: A Pathway to Potent Antifungal Agents
Source: J Med Chem. 2025 Apr 22;68(9):9623–37. doi: 10.1021/acs.jmedchem.5c00396 (PMC12067429; doi:10.1021/acs.jmedchem.5c00396)
Supplement: Supplementary file 1 — jm5c00396_si_001.pdf [file jm5c00396_si_001.pdf]

Supporting Information

for the article

On-resin assembly of macrocyclic inhibitors of Cryptococcus neoformans May1: A pathway to potent antifungal agents

Robin Kryštůfek[1,2], Václav Verner[1], Pavel Šácha[1,3], Martin Hadzima[1,†], Filip Trajhan[1,4], Jana Starková[1], Eva Tloušťová[1], Alexandra Dvořáková[1], Adam Pecina[1], Jiří Brynda[1,5], Karel Chalupský[1], Miroslav Hájek[1], Michael J. Boucher[6], Pavel Majer[1], Jan Řezáč[1], Hiten D. Madhani[6], Charles S. Craik[7], Jan Konvalinka[1,3]*

[1] Institute of Organic Chemistry and Biochemistry of the Czech Academy of Sciences, Flemingovo n. 2, Prague 6 16610, Czech Republic

[2] Department of Physical and Macromolecular Chemistry, Faculty of Science, Charles University, Hlavova 8, Prague 2 12843, Czech Republic

[3] Department of Biochemistry, Faculty of Science, Charles University, Hlavova 8, Prague 2 12843, Czech Republic

[4] Department of Organic Chemistry, Faculty of Science, Charles University, Hlavova 8, Prague 2 12843, Czech Republic

[5] Institute of Molecular Genetics of the Czech Academy of Sciences, Vídeňská 1083, Prague 4 14220, Czech Republic

[6] Department of Biochemistry & Biophysics, University of California, San Francisco, UCSF Genentech Hall, 600 16th St Rm N374, San Francisco, California 94158, United States

[7] Department of Pharmaceutical Chemistry, University of California San Francisco, UCSF Genentech Hall, 600 16th St Rm S512, San Francisco, California 94158, United States

† Current address: Adalid Sciences, Podbabská 30, Prague 6 16000, Czech Republic

* Email: konval@uochb.cas.cz. Phone: +420 220 183 218

Table of contents

Syntheses of aliphatic amino acids S2

Table S1 .... S5

Table S2 .... S6

Table S3 .... S7

Table S4 .... S8

Table S5 .... S9

Table S6 .... S10

Table S7 .... S11

Table S8 .... S12

Table S9 .... S13

Figure S1 .... S14

Figure S2 .... S15

Figure S3 .... S16

Figure S4 .... S17

Figure S5 .... S18

Supplementary references S19

Syntheses of aliphatic amino acids

The synthesis of 13-aminotridecanoic acid (**S1**) involved a four-step process (Scheme S1) starting with tridecane-1,13-diol, which undergoes bromination with hydrobromic acid. This intermediate reacts with potassium phthalimide to form a phthalimide-protected derivative. Subsequent oxidation using Oxone and 2-iodobenzoic acid produces the corresponding carboxylic acid, which is then hydrolysed to yield **S1** with a 44% yield over four steps.

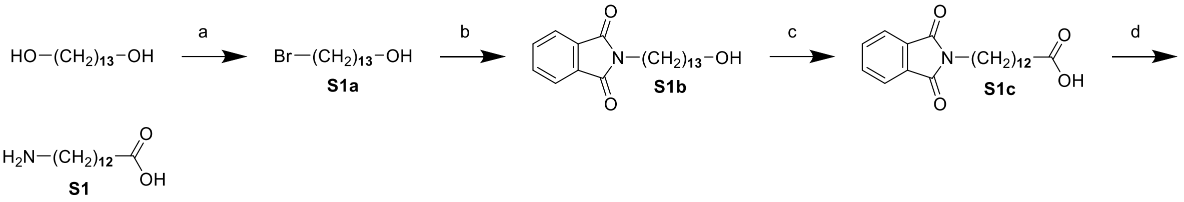

Scheme S1. Synthesis of 13-aminotridecanoic acid **S1**. (a) HBr (aq.), Cyclohexane, reflux, 5 h (b) Potassium phthalimide, DMF, 60 °C, 24 h (c) Oxone, 2-iodobenzoic acid, ACN/water 2:1, 70 °C, 7 h (d) 1. NaOH (aq.), ACN/water 1:1, 70 °C, 1 h; 2. HCl (aq.), 70 °C, 2 h

15-aminopentadecanoic acid (**S2**) was prepared following a modified published procedure[S1] via Beckmann rearrangement and hydrolysis of the resulting lactam (Scheme S2) with a 63% yield over two steps.

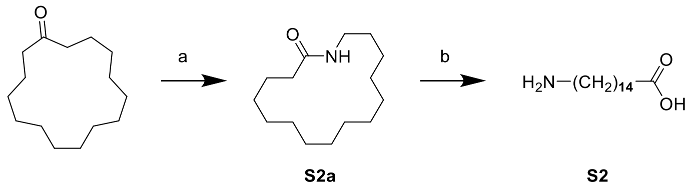

Scheme S2. Synthesis of 15-aminopentadecanoic acid **S2**. (a) H_2NOSO_3H, HCOOH, reflux, 16 h (b) HCl (aq.), reflux, 40 h

13-bromotridecan-1-ol (S1a)
Tridecane-1,13-diol (2 g, 9.24 mmol) was suspended in cyclohexane (30 ml) and concentrated hydrobromic acid (30 ml) was added. The reaction mixture was heated to reflux and stirred for 5 h. Layers were separated and the product was extracted with hexane (3 × 60 ml). Organic layers were combined, washed with saturated $NaHCO_3$ (4 × 40 ml), brine (100 ml), dried over Na_2SO_4 and the organic solvent was removed *in vacuo*. Residue was dissolved in EtOAc and adsorbed onto a silica gel pad, which was washed with hexane (300 ml) and eluted with EtOAc (300 ml). The organic solvent was evaporated to afford the desired product **S1a** (1.47 g, 57%) as colorless crystals.
^{1}H NMR (401 MHz, CD_3CN) δ 1.25 – 1.36 (m, 18H), 1.35 – 1.52 (m, 2H), 1.77 – 1.89 (m, 2H), 2.47 (t, *J* = 5.0 Hz, 1H), 3.42 – 3.51 (m, 4H).
^{13}C NMR (101 MHz, CD_3CN) δ 26.64, 28.80, 29.42, 30.15, 30.22, 30.25, 30.31, 30.33, 30.38, 33.61, 33.63, 35.39, 62.62.
ESI MS: 318 ($[M + K]^+$).

2-(13-hydroxytridecyl)isoindoline-1,3-dione (S1b)
Compound **S1a** (1.357 g, 4.86 mmol) was dissolved in DMF (7 ml) and potassium phthalimide (1.081 g, 5.84 mmol) was added. The reaction mixture was stirred at 60 °C for 24 h. The reaction mixture was diluted with water (40 ml) and extracted with EtOAc (3 × 60 ml). Organic layers were combined, washed with brine (40 ml), dried over Na_2SO_4 and the organic solvent was removed *in vacuo*. The residue was chromatographed on C_{18}-reversed phase silica gel (gradient 0.1% TFA in H_2O – ACN 40%→100%) to obtain the desired product **S1b** (1.629 g, 97%) as an amorphous solid.
^{1}H NMR (401 MHz, $CDCl_3$) δ 1.18 – 1.43 (m, 19H), 1.50 – 1.61 (m, 2H), 1.61 – 1.72 (m, 1H), 3.59 – 3.71 (m, 4H), 7.70 (dd, *J* = 5.5, 3.0 Hz, 2H), 7.83 (dd, *J* = 5.4, 3.0 Hz, 2H).
^{13}C NMR (101 MHz, $CDCl_3$) δ 25.84, 26.97, 28.71, 29.29, 29.52, 29.56, 29.63 (2C), 29.66, 29.68, 32.93, 38.21, 63.21, 123.27, 132.30, 133.95, 168.60.

ESI MS: 346 ([M + H]$^+$).
HR ESI MS: calcd for $C_{21}H_{31}O_3NNa^+$ 368.21962; found 368.21967.

13-(1,3-dioxoisoindolin-2-yl)tridecanoic acid (S1c)
Compound **S1b** (1.43 g, 4.14 mmol) was suspended in ACN (36 ml) and H_2O (18 ml) and 2-iodobenzoic acid (327 mg, 0.66 mmol) and Oxone (3.504 g, 5.70 mmol) were added. The reaction mixture was stirred at 70 °C for 7 h. The reaction mixture was cooled down to 0 °C and formed precipitate was filtered, washed with H_2O (2 × 20 ml) and DCM (2 × 20 ml). The filtrate was extracted with DCM (2 × 40 ml) and the organic layers were combined, washed brine (20 ml) and dried over Na_2SO_4. The organic solvent was removed *in vacuo* to afford the desired product **S1c** (1.414 g, 95%) as white amorphous solid.
^{1}H NMR (400 MHz, CD_3OD) δ 1.26 – 1.35 (m, 16H), 1.52 – 1.72 (m, 4H), 2.27 (t, *J* = 7.4 Hz, 2H), 3.61 – 3.71 (m, 2H), 7.74 – 7.89 (m, 4H).
^{13}C NMR (101 MHz, CD_3OD) δ 26.09, 27.84, 29.44, 30.17, 30.22, 30.40, 30.54, 30.57, 30.58, 30.62, 34.94, 38.80, 124.05, 133.36, 135.33, 169.86, 177.70.
ESI MS: 360 ([M + H]$^+$).
HR ESI MS: calcd for $C_{21}H_{28}O_4N^-$ 358.20238; found 358.20206.

13-aminotridecanoic acid (S1)
Compound **S1c** (1.28 g, 3.56 mmol) was suspended in ACN (30 ml) and H_2O (30 ml) and then solution of NaOH (314 mg, 7.84 mmol) in H_2O was added. The reaction mixture was stirred at 70 °C for 1 h. Then concentrated hydrochloric acid (1.2 ml) was added and the reaction mixture was stirred at 70 °C for 2 h. The solvent was removed *in vacuo*. The residue was chromatographed on C_{18}-reversed phase silica gel (gradient 0.1% TFA in H_2O – ACN 0%→60%) to obtain the desired product **S1** (678 mg, 83%) as an amorphous white solid.
^{1}H NMR (400 MHz, d_6-DMSO) δ 1.17 – 1.33 (m, 16H), 1.41 – 1.57 (m, 4H), 2.18 (t, *J* = 7.4 Hz, 2H), 2.75 (t, *J* = 7.6 Hz, 2H).
^{13}C NMR (101 MHz, d_6-DMSO) δ 24.54, 25.81, 26.97, 28.54, 28.59, 28.78, 28.85, 28.93, 28.94, 29.01, 33.72, 38.79, 174.53.
ESI MS: 230 ([M + H]$^+$).
HR ESI MS: calcd for $C_{13}H_{26}O_2N^-$ 228.19690; found 228.19706.

Azacyclohexadecan-2-one (S2a)
Compound **S2a** was prepared according to a published procedure[S1]. Cyclopentadecanone (2.50 g, 11.1 mmol) was suspended in HCOOH (12.5 ml) and then a solution of hydroxylamine-*O*-sulfonic acid (1.90 g, 16.7 mmol) in HCOOH (12.5 ml) was added. The reaction mixture was heated to reflux and stirred for 16 h. Reaction mixture was diluted with H_2O (100 ml) and extracted by 3 × 15 ml EtOAc. Combined organic phases were washed with 5% $NaHCO_3$ (5 × 15 ml) and brine (3 × 10 ml). Solvent was removed *in vacuo* and the residue was chromatographed on silica gel (EtOAc) to obtain the desired product **S2a** (2.39 g, 90%) as an amorphous white solid.
^{1}H NMR (401 MHz, $CDCl_3$) δ 1.23 – 1.38 (m, 20H), 1.45 – 1.59 (m, 2H), 1.61 – 1.72 (m, 2H), 2.17 – 2.25 (m, 2H), 3.34 (q, *J* = 5.9 Hz, 2H), 5.58 (s, 1H).
^{13}C NMR (101 MHz, $CDCl_3$) δ 25.53, 25.63, 25.77, 25.83, 25.90, 26.41, 27.13, 27.16, 27.30, 27.69, 27.93, 29.28, 36.86, 39.08, 173.48.
ESI MS: 240 ([M + H]$^+$).
HR ESI MS: calcd for $C_{15}H_{30}ON^+$ 240.23219; found 240.23210.

15-aminopentadecanoic acid (S2)
Compound **S2** was prepared using a modified published procedure[S1]. Compound **S2a** (2.28 g, 9.50 mmol) was suspended in 6M HCl (85 ml). The reaction mixture was heated to reflux and stirred for 40 h. Upon cooling, the formed white precipitate was filtered, resuspended in H_2O (55 ml), and the pH was adjusted to 6 using saturated $NaHCO_3$ solution. Suspension was filtered once again, and the precipitate was washed with ice-cold H_2O (2 × 50 ml). Precipitate was dried *in vacuo* to a constant weight to obtain the desired product **S2** (1.72 g, 70%) as an amorphous white solid.
^{1}H NMR (401 MHz, d_6-DMSO) δ 1.05 – 1.35 (m, 20H), 1.40 – 1.59 (m, 4H), 2.16 (t, *J* = 7.3 Hz, 2H), 2.64 – 2.77 (m, 2H), 8.06 (s, 2H).

^{13}C NMR (101 MHz, d_6-DMSO) δ 24.94, 26.32, 27.35, 29.00 (2C), 29.19, 29.30, 29.36, 29.39, 29.44, 29.48 (2C), 34.14, 39.14, 174.92.
ESI MS: 258 ($[M + H]^+$).
HR ESI MS: calcd for $C_{15}H_{32}O_2N^+$ 258.24276; found 258.24258.

Table S1. Data collection and refinement statistics for May1–**25** complex.

**Crystal data**	
Space group	$C\,2\,2\,2_1$
a, b, c (Å)	97.17 113.39 91.22
α, β, γ (°)	90.00, 90.00, 90.00
Molecules per asymmetric unit	1
Matthews coefficient ($Å^3$ Da^{-1})	3.41
Solvent content (%)	63.96
Max. Resolution (Å)	1.81
**Data collection and processing**	
Wavelength (Å)	1.54187
Resolution limits (Å)	50.0-1.8 (1.86-1.81)
No. of observed reflections	165989 (10398)
No. of unique reflections	44101 (2849)
Multiplicity	3.7 (3.6)
$R_{meas.}$	0.202 (1.76)
R_{merge}[a]	0.175 (1.53)
$CC_{1/2}$[b]	0.991 (0.357)
Completeness (%)	95.8 (84.5)
<I/σI>	6.6 (0.88)
**Refinement statistics**	
Resolution (Å)	73.8-1.8 (1.86-1.81)
Reflections used	42553(2726)
R_{work}[c] (%)	18.5(41.8)
R_{free}[d] (%)	20.2(42.0)
Average B-factor ($Å^2$)	26.6
**Ramachandran plot**	
Most favored regions[e] (%)	96.6
Additional allowed regions[e] (%)	3.4
Disallowed regions[e] (%)	0.0
PDB code	6R61

Values in parentheses report the values in the highest resolution shell.

[a] $R_{merge} = \Sigma_{hkl} \Sigma_i |I_i(hkl)_i - \langle I(hkl)\rangle| / \Sigma_{hkl} \Sigma_i I_i(hkl)_i$.

[b] $CC_{(1/2)}$ is the correlation coefficient between random half data sets and from its value the Pearson correlation coefficient of the true level of signal can be calculated[S2]:

$$CC^{*} = \sqrt{2\frac{CC_{\frac{1}{2}}}{1} + CC_{\frac{1}{2}}}$$

[c] R-value = $||F_o| - |F_c|| / |F_o|$, where F_o and F_c are the observed and calculated structure factors, respectively.

[d] R_{free} is equivalent to the R-value but is calculated for 5% of the reflections chosen at random and omitted from the refinement process [S3].

[e] As determined by MolProbity [S4].

Table S2. Evaluation of two possible protonation states of the May1–**25** complex. SQM-based calculations indicate that the Asp238 protonation state is the more stable variant, as determined by total stabilization energy of the complex at SQM level (Total SQM), the SQM2.20 score as an affinity predictor, and total interaction 'free' energy (E_{int}). Other terms, including total stabilization energy of the protein (protein_e) and total QM/MM energy of the complex, are provided for comparison.

**Protein variant**	**Energies with no significant water molecules (kcal/mol)**				
	**protein_e**	**Total SQM**	**Total QMMM**	**SQM2.20**	**E_{int}**
Asp40 (OD1 down)	-2615.3	-7984.8	-2927.2	-53.7	-63.0
Asp238 (OD2 up)	-2617.1	-7998.9	-2935.8	-61.2	-71.8
**Difference**	**-1.8**	**-14.1**	**-8.5**	**-7.5**	**-8.8**

**Protein variant**	**Energies with significant Wat175 (kcal/mol)**				
	**protein_e**	**Total SQM**	**Total QMMM**	**SQM2.20**	**E_{int}**
Asp40 (OD1 down)	-2626.5	-8046.3	-2940.4	-57.6	-67.1
Asp238 (OD2 up)	-2629.8	-8064.5	-2951.0	-65.0	-75.9
**Difference**	**-3.3**	**-18.1**	**-10.5**	**-7.4**	**-8.9**

Table S3. Comparison of the binding affinity of **25** and **21**, as estimated by the SQM-based scoring methodology, in the protein with or without a water molecule, confirming **25** as the more potent inhibitor.

**P1′ modification**	**No significant water**		**With Wat175**		**K_i (nM)**
	**SQM2.20 (kcal/mol)**	**E_{int} (kcal/mol)**	**SQM2.20 (kcal/mol)**	**E_{int} (kcal/mol)**	
**Compound 25 (3Pal)**	-61.2	-71.8	-65.0	-75.9	0.18 ± 0.15
**Compound 21 (Val)**	-56.7	-70.8	-56.7	-70.8	3.6 ± 1.1
**Difference**	**-4.5**	**-1.1**	**-8.3**	**-5.2**	**n/a**

Table S4. Total interaction 'free' energy (E_{int}), interaction energy in the gas phase (Vacuo), and desolvation penalty (Solvation) calculated by the SQM2.20 scoring function for **25** without specific ligand fragments.

**Fragment**	**E_{int} (kcal/mol)**	**Vacuo (kcal/mol)**	**Solvation (kcal/mol)**
**P1b**	-50.6	-122.1	71.6
**P1-P1′**	-63.0	-134.6	71.6
**P1′**	-66.9	-136.1	69.2
**P1′-P2′**	-66.1	-131.4	65.4
**bridge**	-57.2	-138.1	80.9
**P1-P2**	-56.9	-127.8	71.0
**P1a**	-61.6	-135.1	73.5
**Compound 25**	-75.9	-152.9	77.0

Table S5. Energy contributions to the total interaction 'free' energy (E_{int}), interaction energy in gas phase (Vacuo) and desolvation penalty (Solvation) calculated by SQM2.20 scoring function for individual compound **25** fragments. E_{int} represents the interaction 'free' energy change upon binding, calculated as the sum of the gas-phase interaction energy and the desolvation energy term. The fragment contributions are obtained by systematically deleting each fragment from **25** and capping the remaining molecule with hydrogen atoms. This approach introduces slight inconsistencies, making the fragment contributions not perfectly additive.

Fragment	Contribution (kcal/mol)		
	E_{int}	Vacuo	Solvation
**P1b**	25.3	30.7	-5.4
**P1-P1′**	12.9	18.3	-5.3
**P1′**	9.0	16.8	-7.8
**P1′-P2′**	9.9	21.4	-11.6
**Bridge**	18.7	14.8	3.9
**P1-P2**	19.1	25.1	-6.0
**P1a**	14.3	17.8	-3.5
**Compound 25**	75.9	152.9	-77.0
**Sum**	105.6	141.5	-35.9

Table S6. Plasma stability of **9** and **25**, evaluated by incubating 5 μM solutions with human pooled plasma at 37 °C over a period of 2 h. Propantheline is included as a positive control for plasma instability.

**Compound**	**Human**		**Mouse**	
	**$T_{1/2}$ (min)**	**Remaining at 120 min (%)**	**$T_{1/2}$ (min)**	**Remaining at 120 min (%)**
**9**	stable	95.3 ± 13.5	294 ± 29	66.0 ± 6.2
**25**	stable	87 ± 5	stable	104 ± 8
Propantheline	*13.7 ± 0.8*	*0.2 ± 0.1*	*36.3 ± 0.6*	*12.9 ± 0.6*

Table S7. Microsomal stability of **9** and **25**, evaluated by incubating 5 μM solutions with pooled microsomes at 37 °C over a period of 45 min. Verapamil is included as a positive control for microsomal instability.

**Compound**	**Human**			**Mouse**		
	**$T_{1/2}$ (min)**	**Remaining at 45 min (%)**	**CL_{int} (μl/min/mg)**	**$T_{1/2}$ (min)**	**Remaining at 45 min (%)**	**CL_{int} (μl/min/mg)**
**9**	stable	102.6 ± 8.9	N/A	stable	108.7 ± 7.3	N/A
**25**	44 ± 4	47 ± 6	32 ± 3	35 ± 8	42 ± 12	41 ± 9
Verapamil	*19.4 ± 1.0*	*20.9 ± 0.8*	*71.9 ± 3.6*	*12.4 ± 0.2*	*7.7 ± 0.3*	*113.1 ± 0.8*

Table S8. Caco-2 permeability assay for **9** and **25** conducted over a 3-h transport period. The efflux ratio was calculated as the ratio of permeability in the basolateral-to-apical direction (P_{app} B→A) to that in the apical-to-basolateral direction (P_{app} A→B).

**Compound**	**A→B**		**B→A**		**Efflux ratio**
	**P_{app} (cm/s) × 10^{-6}**	**Recovery (%)**	**P_{app} (cm/s) × 10^{-6}**	**Recovery (%)**	
**9**	2.9 ± 0.7	125	71 ± 15	84	25
**25**	0.9 ± 0.6	34	33.3 ± 0.8	54	38

Table S9. Pharmacokinetic properties of **9** and **25** in male C57BL6/N mice. Parameters were calculated using PK Solver 2.0 [S5].

Compound	Oral bioavailability	i.v.		p.o.	
		$T_{1/2}$ (min)	c_{max} (ng/ml)	$T_{1/2}$ (min)	c_{max} (ng/ml)
**9**	6.0 %	79.6	26.4	42.4	21.2
**25**	12.5 %	6.9	73.8	83.7	10.4

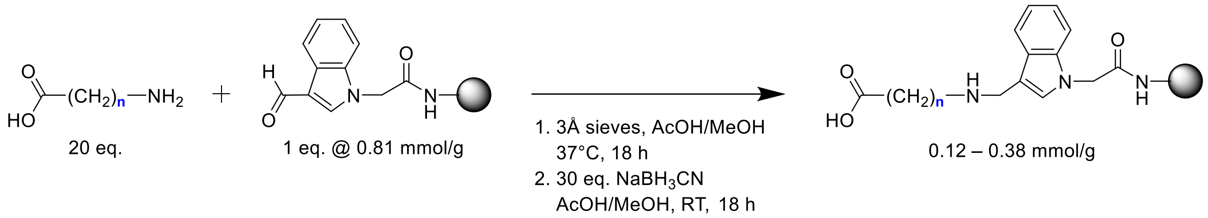

A

n	loading mmol/g	yield
**3**	0.36	45%
**4**	0.38	47%
**5**	0.36	44%
**6**	0.37	46%
**7**	0.21	26%
**8**	0.17	21%
**9**	0.15	19%
**10**	0.12	15%
**11**	0.18	23%
**12**	0.16	20%
**13**	0.09	12%
**14**	0.17	21%
**15**	0.22	27%

	loading mmol/g
**average**	0.23
**s.d.**	0.10
**min**	0.09
**max**	0.38

B

n = 11	dehydration agent (1.)	loading mmol/g	yield
20 eq.	3Å sieves	0.18	23%
10 eq.	3Å sieves	0.14	17%
2 eq.	3Å sieves	0.07	9%
10 eq.	50% TMOF	0.05	6%
2 eq.	50% TMOF	0.02	2%

Figure S1. (a) Loading evaluation for aliphatic amino acids using 3Å molecular sieves as dehydration agent. (b) Comparison of the performance of dehydration agents.

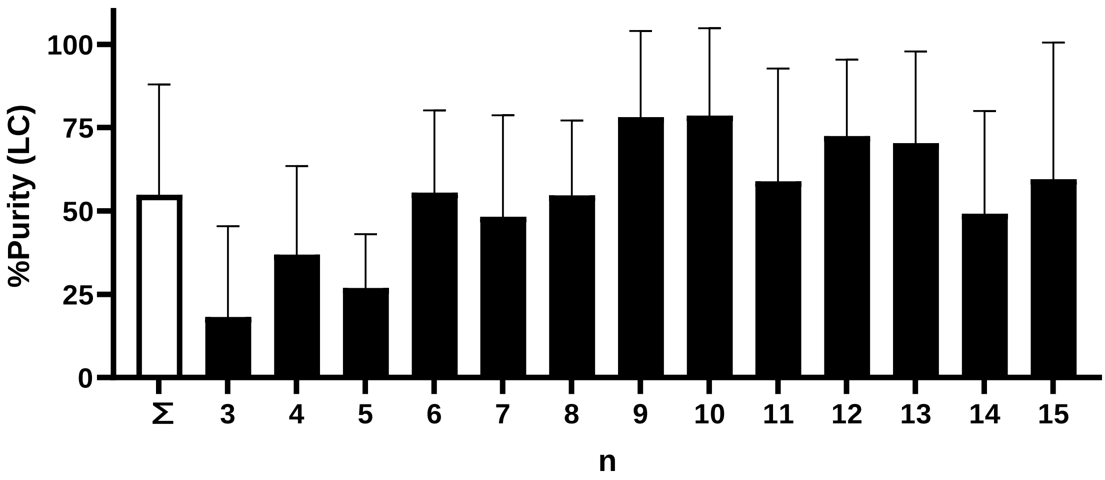

Figure S2. Breakdown of the purity of compounds in the macrocyclic library based on aliphatic linker length (n) as well as total library purity (Σ, for all 13 × 48 = 624 compounds). Purity was analysed using liquid chromatography, with ESI-MS for identity confirmation and ELS detection for quantitation.

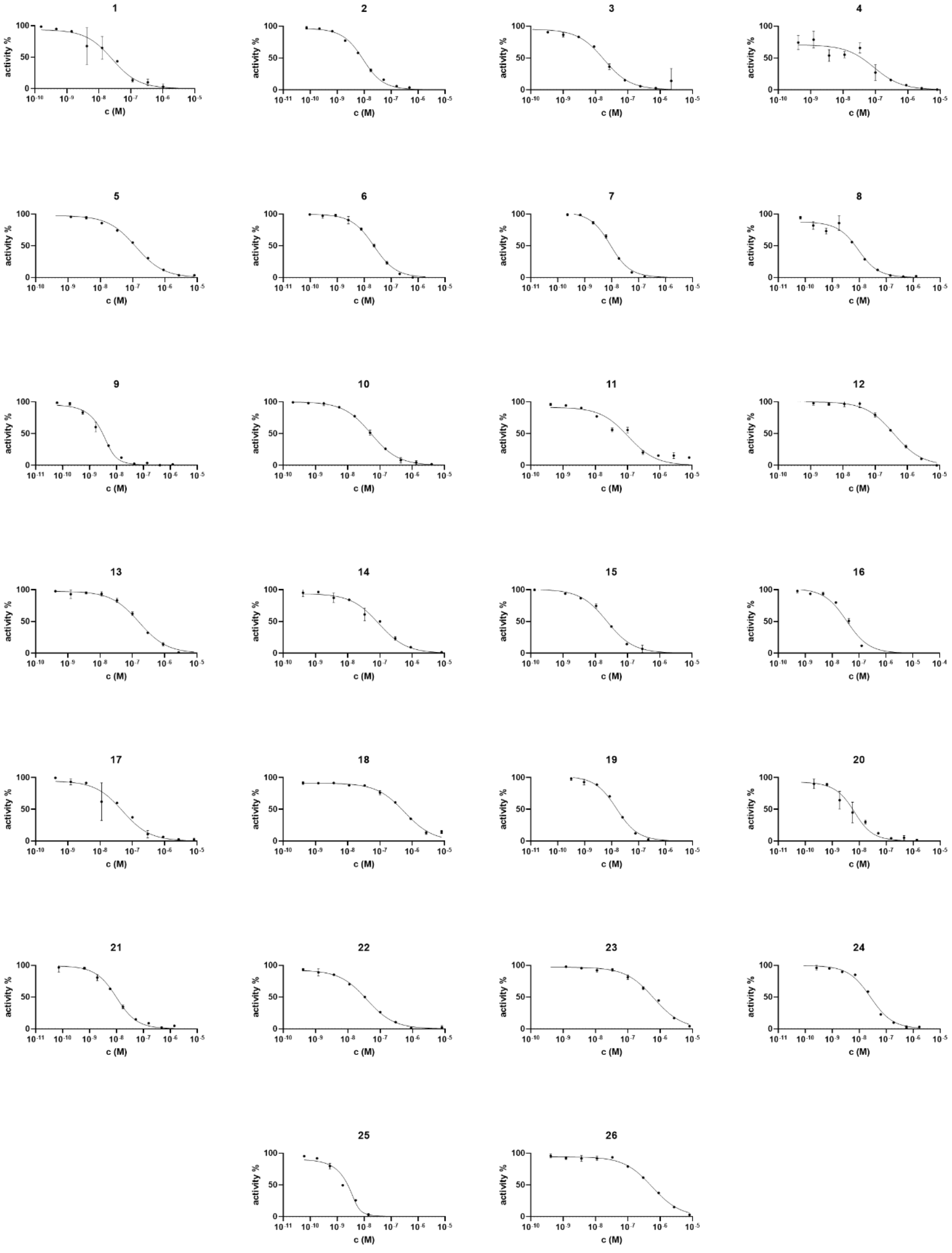

Figure S3. Concentration–response plots for determination of K_i against May1 for compounds **1**–**25** selected for validation.

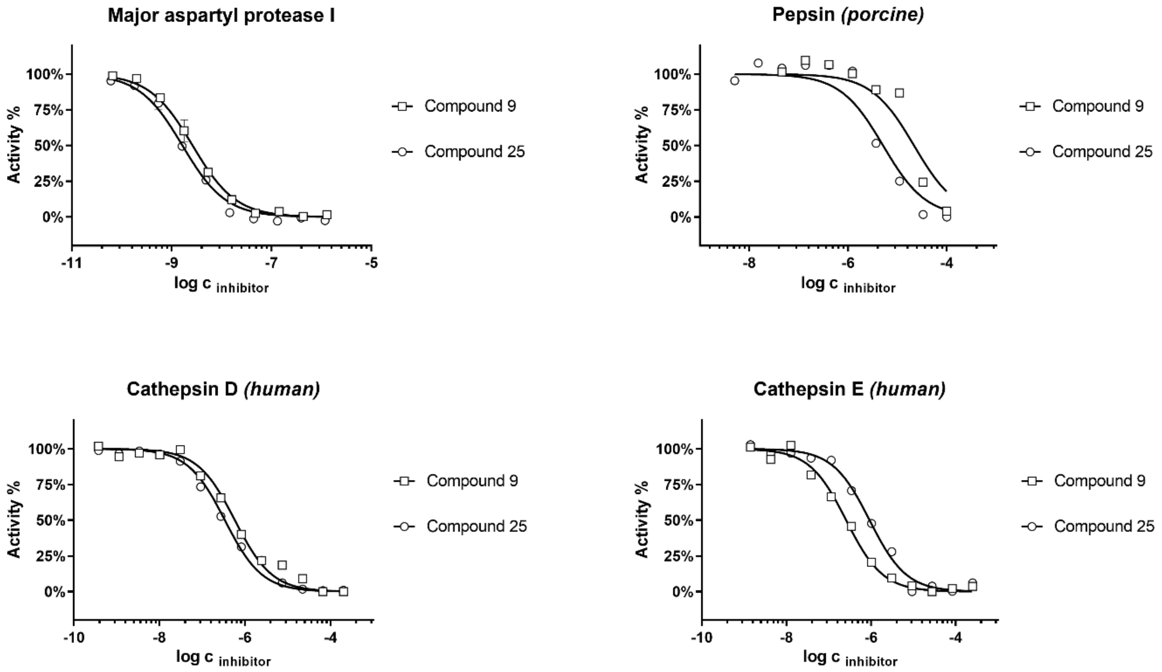

Figure S4. Concentration–response plots for determination of K_i against May1 and selected off-target aspartic proteases for lead compounds.

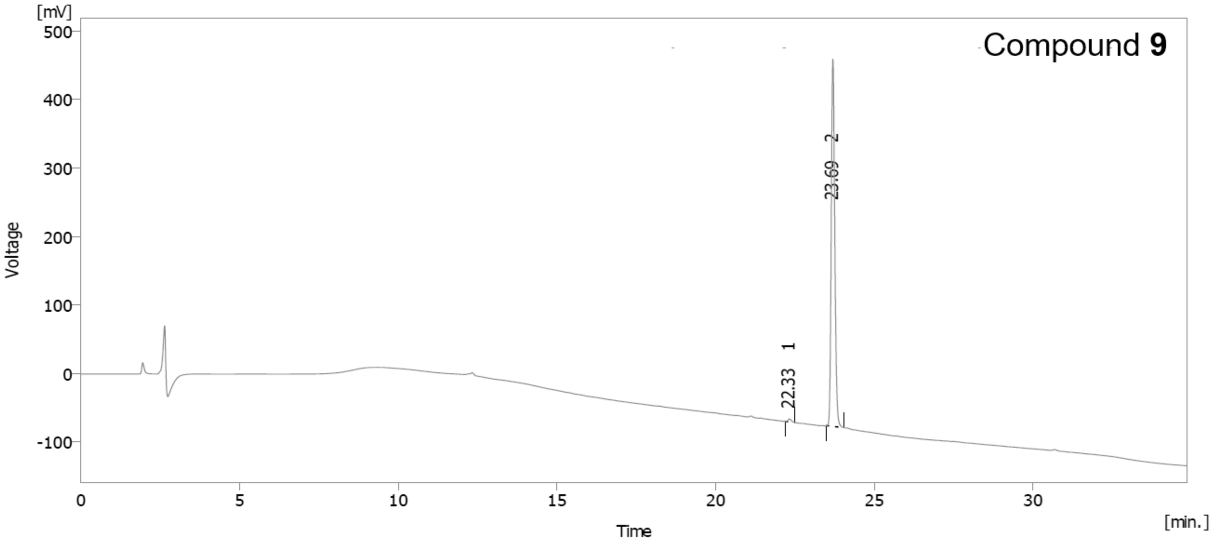

	Reten. Time [min]	Area [%]
1	22.328	0.7
2	23.692	99.3
	Total	100.0

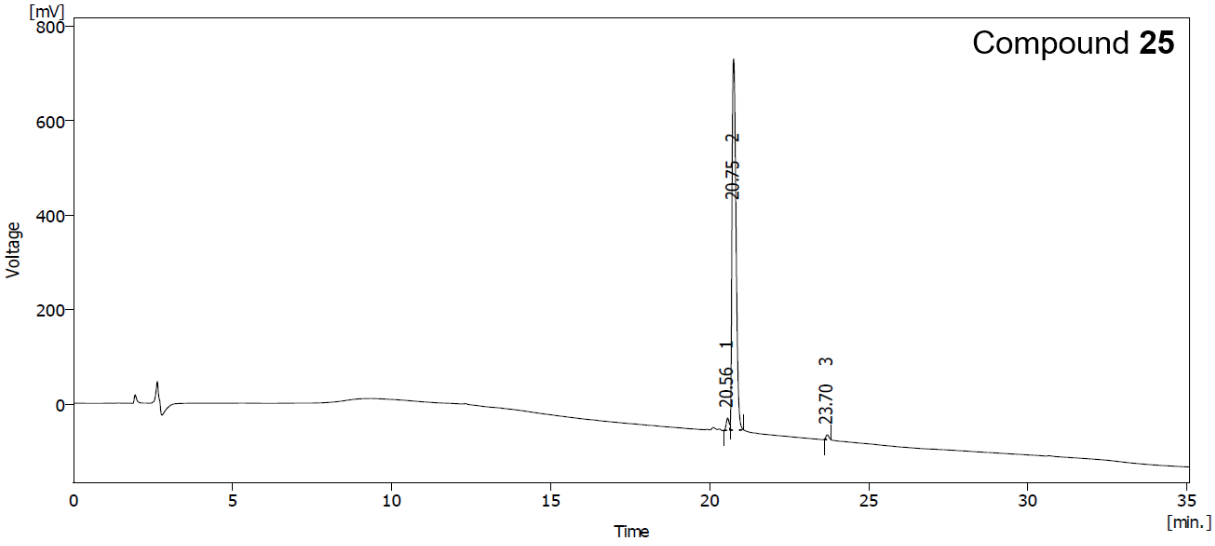

	Reten. Time [min]	Area [%]
1	20.560	2.2
2	20.752	96.9
3	23.696	0.9
	Total	100.0

Figure S5. Analytical RP-HPLC traces for **9** and **25**. For experimental details, see General procedures in the Experimental section.

Supplementary references:

S1 Vozdvizhenskaya, O. A., *et al.* Synthesis and antiherpetic activity of novel purine conjugates with 7,8-difluoro-3-methyl-3,4-dihydro-2H-1,4-benzoxazine. *Chem Heterocycl Compd* **57**, 490-497 (2021).

S2 Karplus, P. A. & Diederichs, K. Linking crystallographic model and data quality. *Science* **336**, 1030-1033 (2012).

S3 Brunger, A. T. Free R value: a novel statistical quantity for assessing the accuracy of crystal structures. *Nature* **355**, 472-475 (1992).

S4 Chen, V. B. *et al.* MolProbity: all-atom structure validation for macromolecular crystallography. *Acta Cryst D* **66**, 12-21 (2010).

S5 Zhang, Y., *et al.* PKSolver: An add-in program for pharmacokinetic and pharmacodynamic data analysis in Microsoft Excel. *Comput Methods Programs Biomed* **99**, 306-314 (2010).
